# Hybrid Inorganic‐Organic Rotaxanes and Related Compounds

**DOI:** 10.1002/chem.202501067

**Published:** 2025-05-30

**Authors:** Selena J. Lockyer, George F. S. Whitehead, Grigore A. Timco, Eric J. L. McInnes, Richard E. P. Winpenny

**Affiliations:** ^1^ Department of Chemistry and Photon Science Institute The University of Manchester Oxford Road Manchester M13 9PL UK

**Keywords:** carboxylate clusters, heterometallic rings, metallocycles, molecular magnets, supramolecular chemistry

## Abstract

The extension of previous studies of heterometallic rings (HMRs) is described, including new synthetic chemistry and physics. These HMRs feature a ring of, typically, eight metal centers with a central charge‐balancing cation. New HMRs and related molecules are described, varying the number of metals present. The eight metal HMRs have been used as components of larger supramolecular assembly, most often by making the HMRs into [2]rotaxanes or pseudo‐rotaxanes, where the thread of the rotaxane is terminated by a binding group such as pyridine. This allows the formation of [n]rotaxanes, where n = 3, 4, 5,7, 13, or 10^14^. Mass spectrometry and NMR studies of the HMRs have given greater understanding of the host‐guest chemistry of these metal analogues of crown ethers. Physical studies have included 4D‐inelastic neutron scattering (INS) and continuous wave and pulsed EPR spectroscopy to measure magnetic interactions between spin components of the supramolecular assemblies. The use of the HMRs as resists for high‐resolution lithography is also discussed.

## Graphical abstract

A review of HRMs over the last 10 years is presented, including aspects of their supramolecular chemistry, magnetic properties, and applications.

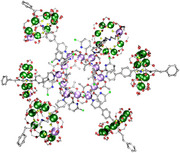

## 1 Introduction

Cyclic compounds are attractive to study from several perspectives. Most mechanically interlocked compounds, whether rotaxanes or catenanes, involve a ring, and therefore organic rings underpin molecular machines.^[^ ^1^, ^2^, ^3^, ^4^ ^]^ When studying magnetic molecules, complexes where the spin centers are arranged in a ring are simpler to model than molecules where the spin centers have more complicated topology.

**Figure 1:**
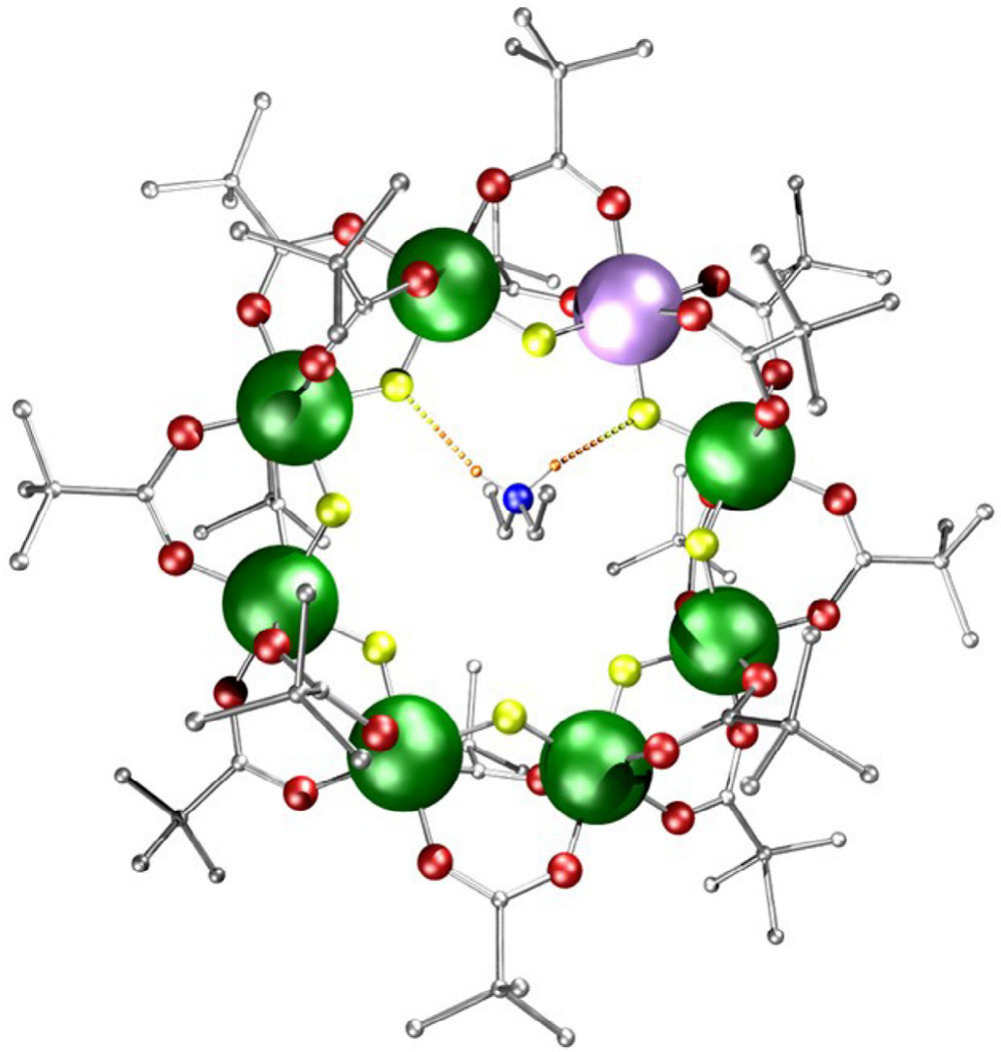
Structure of **1** **M**, where M ═ Ni, the {Cr_7_Ni} ring [Et_2_NH_2_][Cr_7_NiF_8_(O_2_C*^t^* Bu)_16_]. Atom colors: Cr, green; Ni, lilac; O, red; N, blue; F, yellow; C, grey; H, orange. Dashed orange to yellow line representing hydrogen bonding between the ammonium ion and ring. Hydrogens bound to carbons omitted for clarity.

We have been studying heterometallic rings (HMRs) for around twenty years because they lie at the intersection between supramolecular chemistry and molecular magnetism. The first family of such rings was reported in 2003,^[^ ^5^ ^]^ and has the general formula [R_2_NH_2_][Cr^III^_7_M^II^F_8_(O_2_C^t^Bu)_16_] **1** **M** (R ═ Me, Et, or ^n^Pr; M ═ Ni, Co, Zn, Mn, or Cd). We have reviewed the area previously,^[^ ^6^ ^]^ and here we focus on developments in the period since 2015, only referring to earlier work where necessary. These HRMs contain an octagon of metals, with the divalent metal disordered over the eight sites of the ring (Figure 1). Each edge of the octagon is bridged by two carboxylates and one fluoride; the fluorides line the internal cavity of the ring and form hydrogen bonds to the secondary ammonium cation. While the divalent metal site is disordered, there is some preference for it to be found bound to the two fluorides involved in the H‐bonds to the ammonium cation.

The work pursued in that period can be broken into five areas. First, we will introduce work on individual rings or related acyclic chains. Most of the synthetic work has focused on the idea that these HMRs could be used as quantum bits (qubits) within quantum computers. This has led to work studying their assembly into larger supramolecules, and the synthesis of complexes involving multiple spin centers, and we discuss this second. These compounds have then been studied by continuous wave (c.w.) and pulsed EPR spectroscopy to measure the interaction energies between spin centers. This work has also led to a deeper investigation into the chemistry, examining what other HMRs can be made beyond the {Cr^III^_7_M^II^} parent.

The new chemistry has also led to more in‐depth studies of the host‐guest chemistry of these HMRs. We can use both mass spectrometry and NMR spectroscopy to examine how the cation leaves the anionic ring. This forms the third section of this review.

The physics of these HMRs continues to be interesting, and they are also interesting objects to use for other physical studies. Many of these physical studies refer to the overall aim to use the HMRs in quantum computing, but others are concerned with understanding how the anisotropy of the components of a molecular magnet contributes to the overall anisotropy.

In the final section of the review, we examine two possible applications of these HMRs. The first application is as resist materials for lithography, where they are promising for electron beam and extreme UV lithography. The second application looks at the host‐guest chemistry, but now tries to trap small molecules within the cavity of the ring. For this we returned to the original homometallic [CrF(O_2_C^t^Bu)_2_]_8_ ring **2**.

## 2 Developing New Single Rings and Chains

The chemistry we have developed is based on a straightforward one‐pot reaction^[^ ^5^, ^6^ ^]^:

$$\begin{array}{ccc} & & 7\;\mathrm{Cr}F_{3}.4H_{2}O+"{\mathrm{MX}}_{2}"+\mathrm{HN}R_{2}+\mathrm{xs}\;{\mathrm{HO}}_{2}C^{t}\mathrm{Bu}\\ & & \;\to \left[H_{2}{\mathrm{NR}}_{2}\right]\left[{\mathrm{Cr}}_{7}{\mathrm{MF}}_{8}{\left(O_{2}C^{t}\mathrm{Bu}\right)}_{16}\right] \end{array}$$

**Figure 2:**
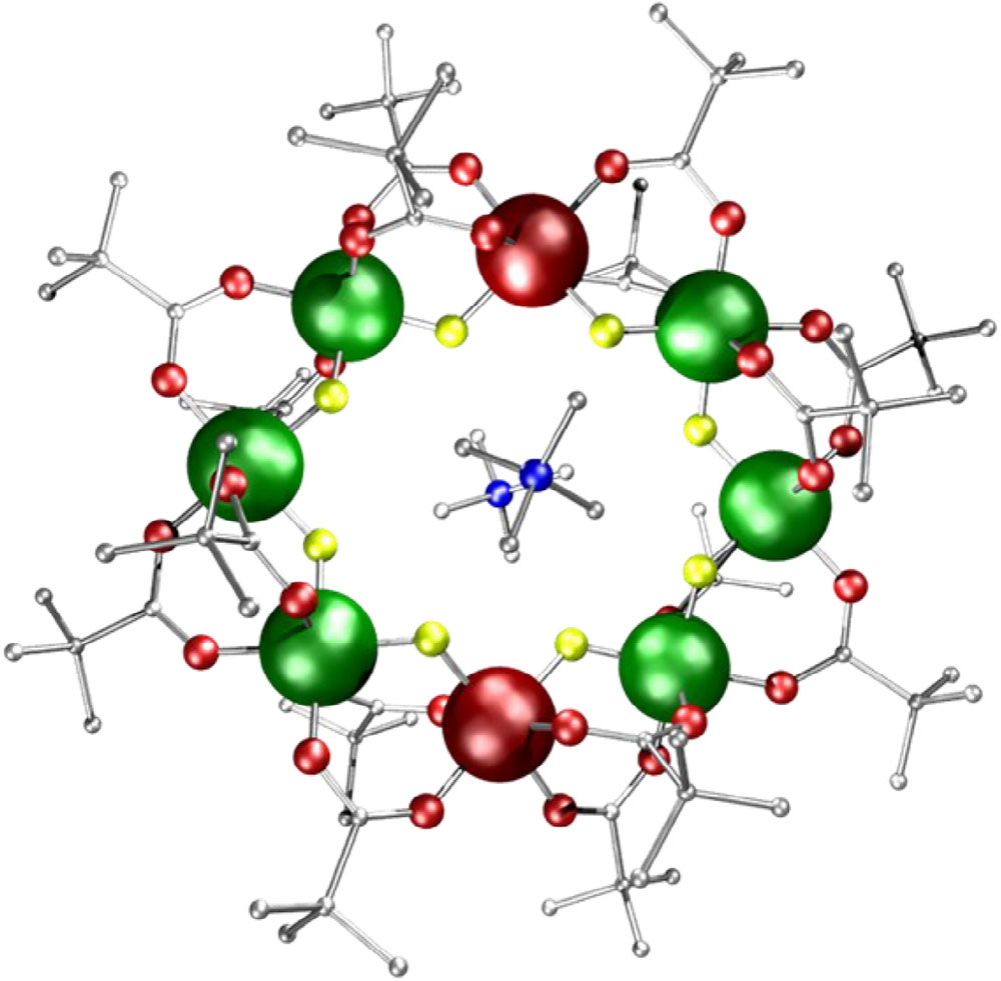
Structure of **3** **M**, [Me_4_N]_2_[Cr_6_M_2_F_8_(O_2_C*^t^* Bu)_16_] where M = Zn. Atom colors as in Figure 1, with Zn, brown. Hydrogens omitted for clarity.

MX_2_ is a divalent metal salt, typically a metal carbonate such as basic nickel or zinc carbonate. R is typically a linear alkyl group. One new variation arises if we use tetramethylammonium as the cation, which removes the possibility of H‐bonding to the inside of the octagonal cavity. In the reaction, HNR_2_ is replaced by Me_4_N.OH and this gives [Me_4_N]_2_[Cr_6_M_2_F_8_(O_2_C^t^Bu)_16_] **3** **M** (M ═ Ni^II^, Zn^II^, Co^II^, Mn^II^).^[^ ^7^ ^]^ The structure is ordered, with the two divalent metals found at the 1,5‐positions of the octagon (Figure 2). The Me_4_N^+^ cations are found above and below the planes of the ring in the crystal structure. For **3Co,** a well‐resolved NMR spectrum confirms that the 1,5‐isomer is predominant, and this gives a ring with D_2_ symmetry, and the NMR spectrum is consistent with high symmetry. Octahedral Co^II^ has a ^4^T_1g_ ground term (approximating to O_h_ symmetry), arising from the (t_2g_)^5^(e_g_)^2^ configuration. It is this orbital degeneracy that results in very fast electron spin relaxation that allows the solution NMR studies of {Cr^III^_8‐x_Co^II^_x_} rings (x = 1 or 2). Previously when we have had two divalent ions in a ring, we have seen isomerism, for example, in imidazolium complexes of {Cr_X_Ni_2_} rings.^[^ ^8^ ^]^ Here the formation of only one isomer is unexpected, and not easily explained.

**Scheme 1:**
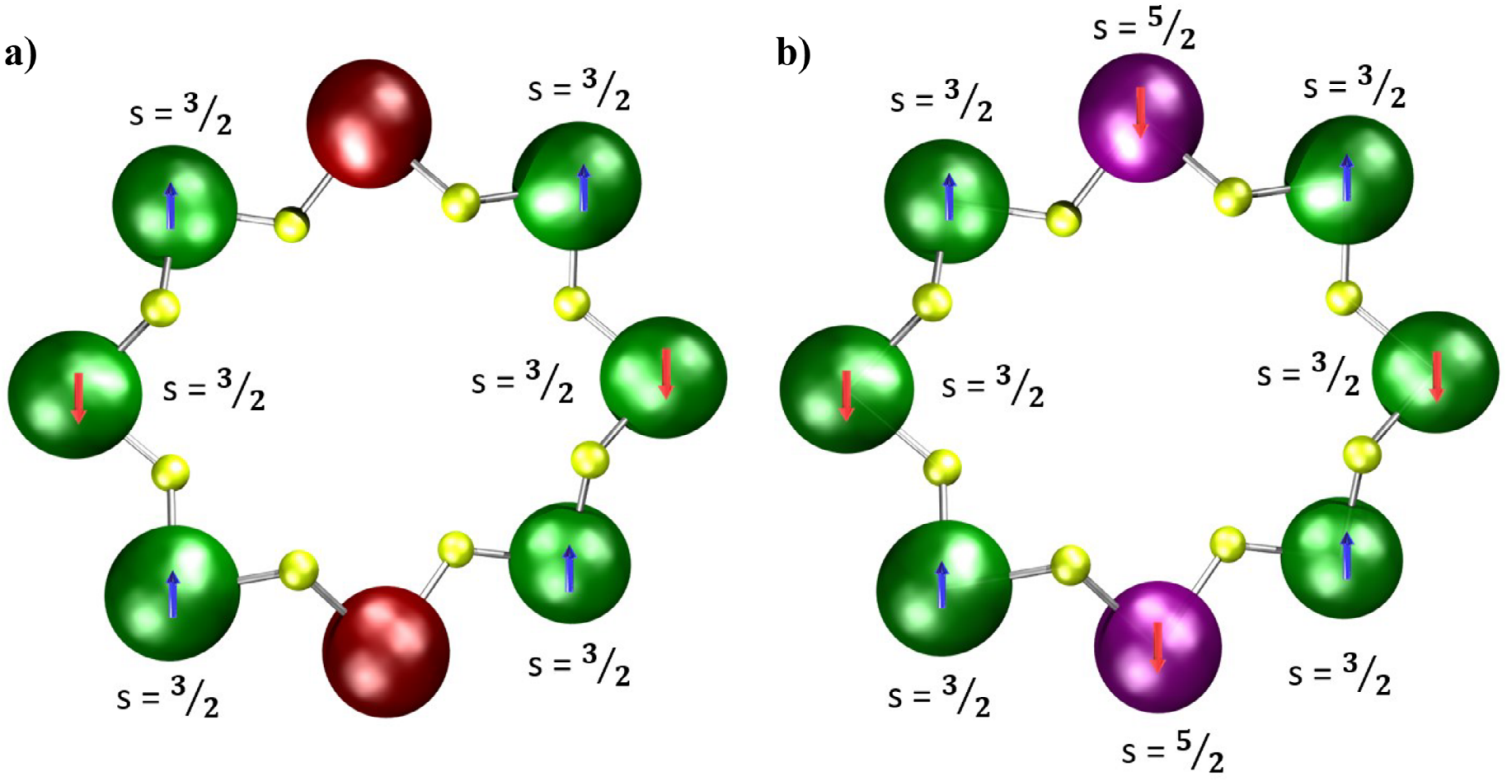
Spin ground states of a) {Cr_6_Zn_2_} and b) {Cr_6_Mn_2_} rings. In a) the diamagnetic Zn^II^ site (brown) gives two *S* = 3/2 chains while in b) the Mn^II^ sites both sit in the same sub‐lattice, giving an *S* = 2 ground state.

EPR and magnetic studies of **3** **Zn** and **3Mn** are consistent with the presence of a single isomer; the EPR of **3** **Zn** can be assigned to *S* = 3/2 chains, which are formed if there are two diamagnetic breaks in the octagon (Scheme 1a). For **3Mn**, the EPR is assigned as due to an *S* = 2 ground state, which indicates that the two *s* = 5/2 Mn^II^ ions are in the same sub‐lattice, and logic suggests that the 1,5‐isomer of the ring is the best explanation (Scheme 1b). The data for **3Ni** are less clear‐cut, mainly as the EPR is poorly resolved.

Single isomers were previously seen by the Saalfrank group in [PPh_4_]{Fe^III^[Fe^III^_2_Mn^II^_4_Cl_6_(L)_6_]} and [PPh_4_]{Mn^II^[Mn^II^_3_In^III^_3_Cl_6_(L)_6_]}, where H_2_L = N‐benzyldiethanolamine.^[^ ^9^ ^]^ In those cases, the argument was made that charge separation favored keeping the trivalent ions as far apart as possible. This could also apply in **3M** which differs from other HRMs in not having a cation at the center of the ring, rather the Me_4_N^+^ cations are above and below the plane of the ring.

**Figure 3:**
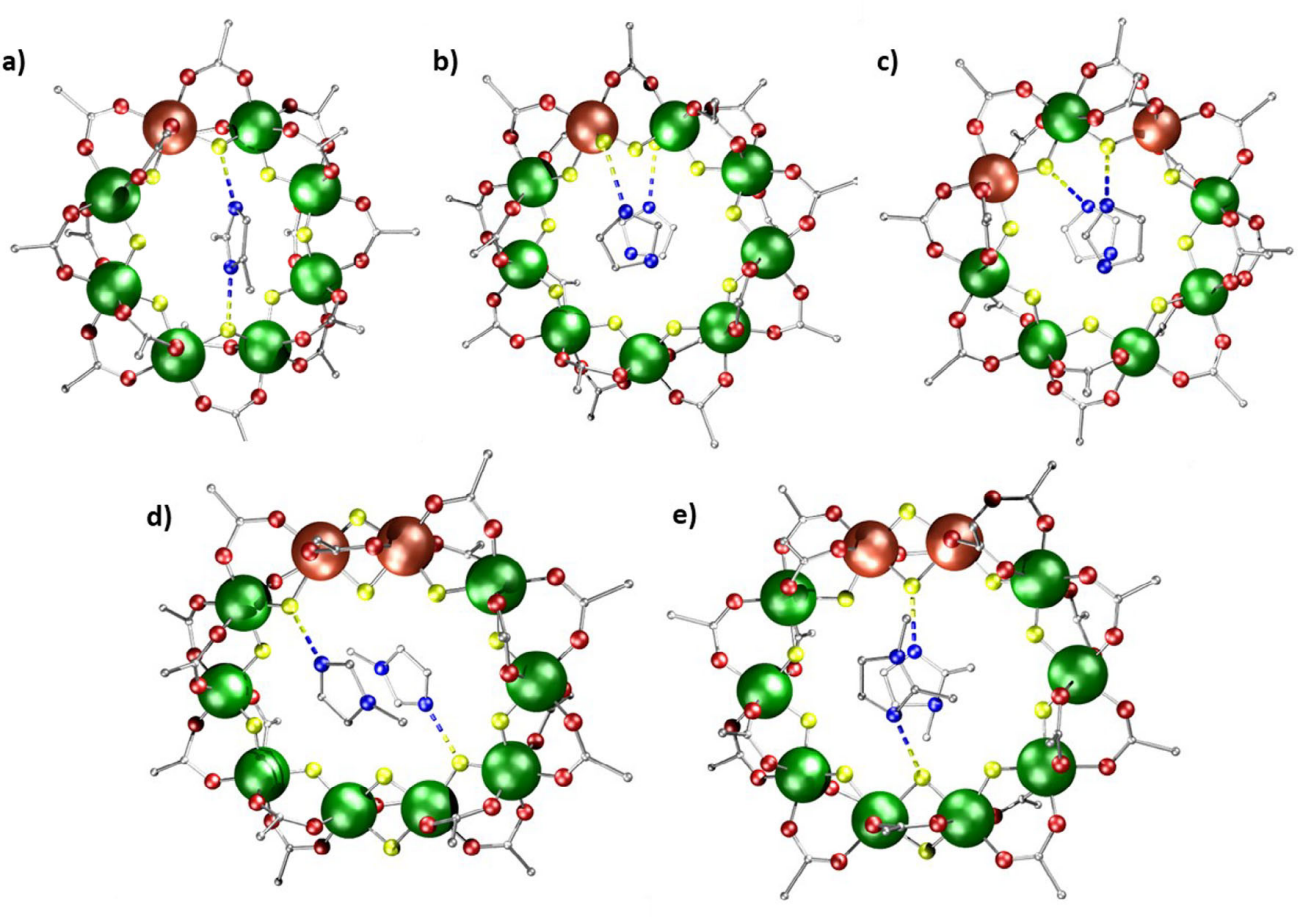
Structures of a) **4**, b) **5**, c) **6**, d) **7,** and e) **8**. Atom colors as Figure 2, with blue and yellow dashed lines represent hydrogen bonding. Hydrogens and methyl groups omitted for clarity.

Use of imidazolium as a templating cation was extended to produce multiple {Cr_x_Zn_y_} complexes.^[^ ^10^ ^]^ [2,4‐DiMe‐ImidH][Cr_7_ZnF_8_(O_2_C^t^Bu)_16_] **4,** [ImidH]_2_[Cr_8_ZnF_11_(O_2_C^t^Bu)_17_] **5**, [ImidH]_2_[Cr_6_Zn_2_F_8_(O_2_C^t^Bu)_16_] **6**, [1‐MeImH]_2_[Cr_8_Zn_2_F_12_(O_2_C^t^Bu)_18_] **7** and [1,2‐diMeImH]_2_[Cr_8_Zn_2_F_12_(O_2_C^t^Bu)_18_] **8**, can be prepared using various imidazoles in the reaction (2,4‐DiMe‐Imid = 2,4‐dimethylimidazole, Imid = imidazole, [1,2‐diMeIm] = 1,2‐dimethylimidazole) (Figure 3).

While **4** and **6** are eightmetal rings, **7** and **8** are ten‐metal rings, and **5** a rare nine‐metal ring. The eightmetal rings have two carboxylates and one fluoride on each edge, as expected. The ten‐metal rings have eight edges bridged in this way, but the final two edges are bridged by two fluorides and one pivalate (Figure 3d and 3e), which leads to a distorted decagon. The nine‐metal ring **5** (Figure 3b) has one edge which has only one fluoride and one pivalate, with terminal fluorides on both metals in this edge. The terminal fluorides H‐bond to the imidazolium cations. The Zn^II^ sites are thought to be in the edges that contain fewer pivalates bound. EPR spectroscopy and magnetic studies show multiple isomers are present in all the compounds with two Zn^II^ sites.

**Figure 4:**
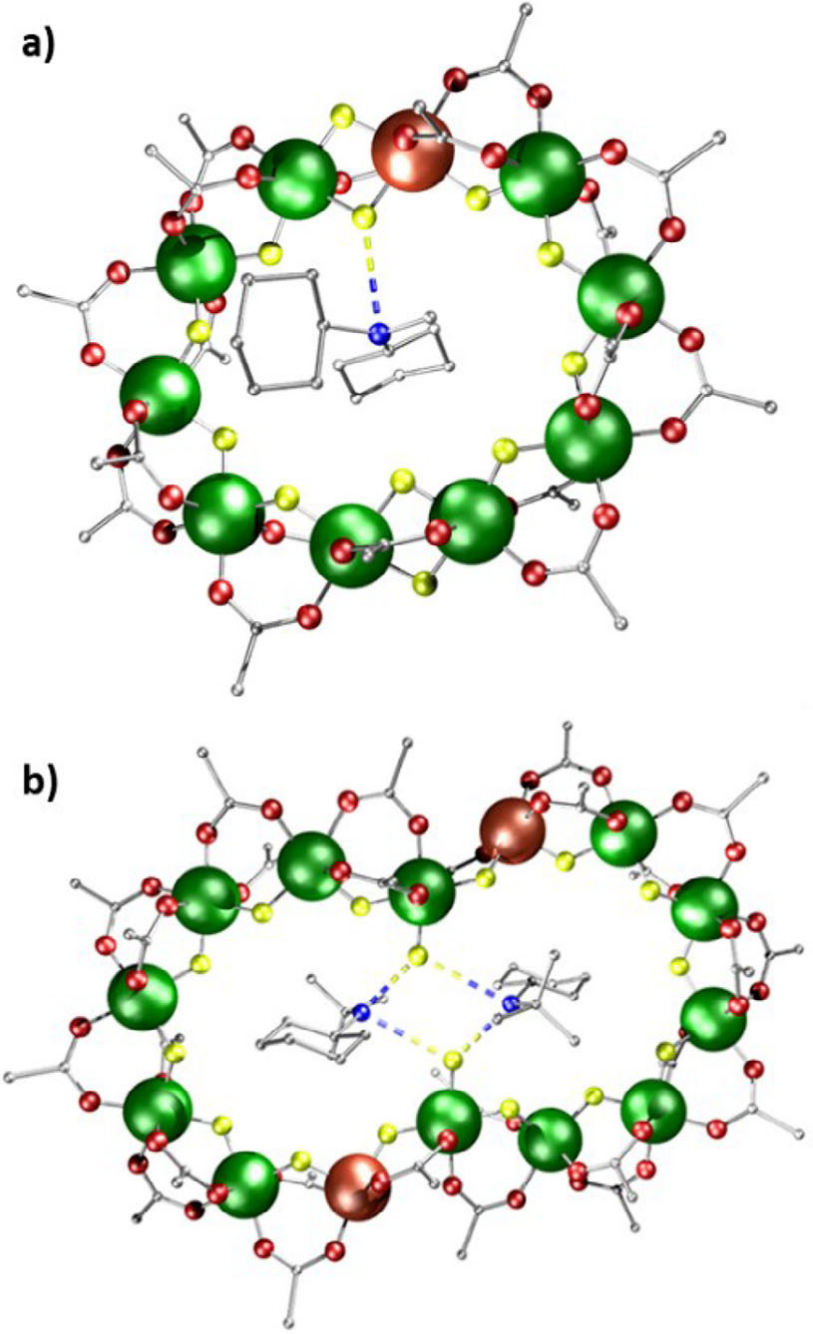
Structures of a) **9** and b) **10**. Atom colors as Figure 3, with blue and yellow dashed lines represent hydrogen bonding. Hydrogens and methyl groups omitted for clarity.

Use of the tertiary amines Cy_2_MeN and Cy_2_ *^t^* BuN in the reaction led to [Cy_2_NHMe][Cr_9_ZnF_12_(O_2_C^t^Bu)_18_] **9** and [CyNH_2_ *^t^* Bu]_2_[Cr_12_Zn_2_F_16_(O_2_C*^t^* Bu)_26_] **10**, respectively.^[^ ^10^ ^]^ Compound **9** is a ten‐metal ring, closely related to **7** and **8** with similar arrangements of ligands around the ring. Compound **10** forms an hourglass of twelve metals, with the two cations found in the centers of each half of the hourglass (Figure 4). The two metals at the waist of the hourglass have terminal fluorides, and these are H‐bonded to the cations. While **10** could have multiple isomers, the combination of X‐ray crystallography, magnetism, and EPR spectroscopy suggests that the structure has the two five‐coordinate Zn^II^ sites separated by {Cr_6_} chains; perhaps most decisive is the EPR spectroscopy, which shows no resonances due to half‐integer spins, which would be found if {Cr_5_} or {Cr_7_} chains were present.

**Figure 5:**
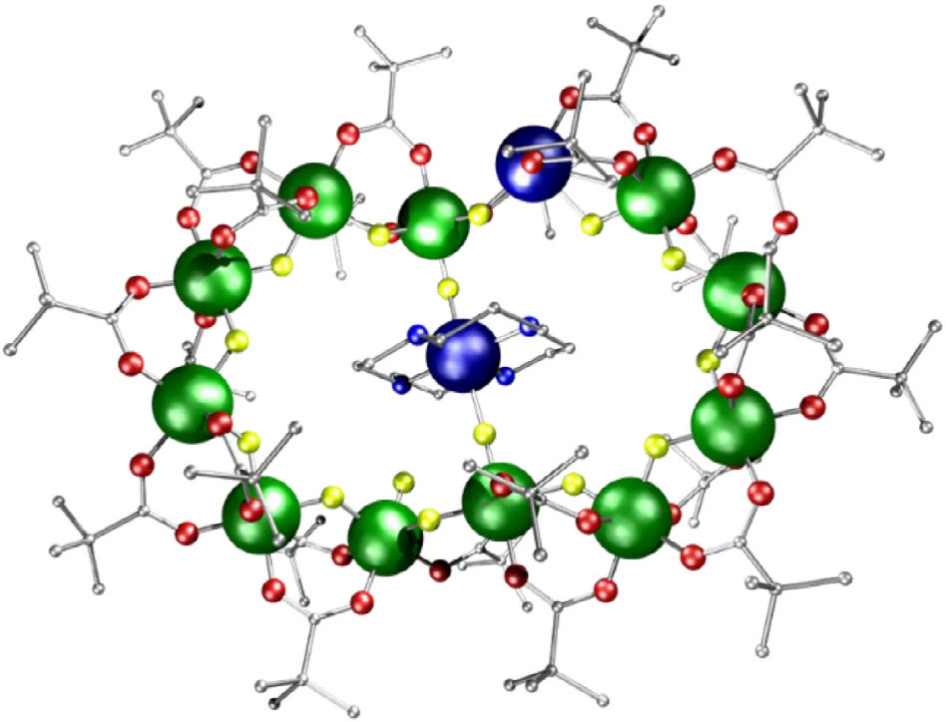
Structures of **11** **M** where M ═ Cu. Atom colors as Figure 1, with Cu dark blue. Hydrogens omitted for clarity.

The final variation which has been used is to include cyclic amines as templates.^[^ ^11^ ^]^ Using 1,4,8,11‐tetrazacyclotetradecane (cyclam) leads to [M(cyclam)][Cr_11_MF_15_(O_2_C^t^Bu)_22_] **11** **M** (M = Zn^II^ or Cu^II^), in which the [M(cyclam)]^2+^ unit is found at the center of a twelve metal ring, which is not as waisted as the hourglass in **10** (Figure 5). Terminal fluorides are again found on the central Cr^III^ sites, but here they bind to the metal from the [M(cyclam)]^2+^ unit. The divalent site within the twelve‐metal ring is five‐coordinate. The number of magnetic centers present leads to the problem that deriving exchange interactions by conventional software, for example PHI,^[^ ^12^ ^]^ becomes impossible because exact numerical diagonalization of the enormous spin Hamiltonian matrices is impossible. Therefore, the magnetic properties were fitted using the Lanczos method, which combines accurate approximations to the eigenvectors (spin states) and eigenvalues (energies) with a random sampling method to calculate thermodynamic properties.^[^ ^13^ ^]^ **11** **Zn** has an *S* = 3/2 ground state. The position for **11Cu** is more complicated due to the bridging paramagnetic metal.

**Figure 6:**
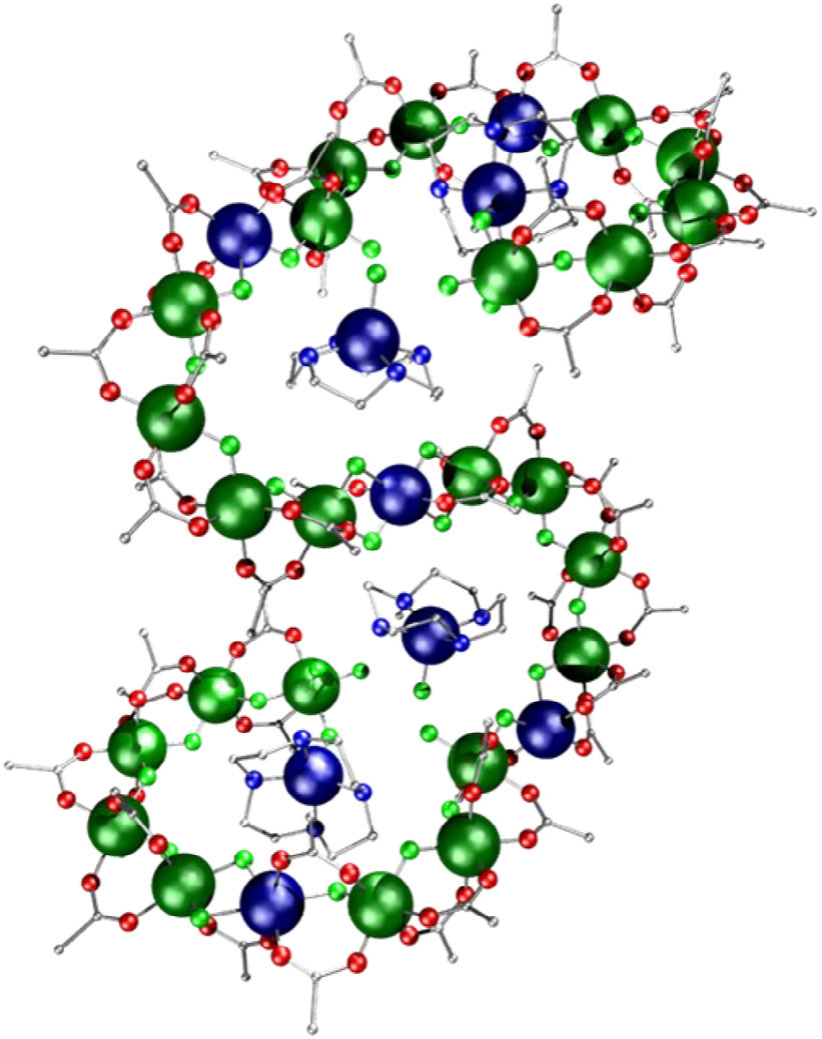
Structure of **12**. Atom colors as Figure 5. Hydrogens and methyl groups omitted for clarity.

The most spectacular complex in this family results from using 1,4,7,10‐tetrazacyclododecane (cyclen) in the reaction with Cu^II^. [Cu(H_2_O) (cyclen)]_2_[Cr_24_Cu_5_{Cu(cyclen)}_2_F_40_(O_2_C^t^Bu)_50_] **12** is formed in low but reproducible yield; **12** is the longest finite paramagnetic chain known (Figure 6).^[^ ^14^ ^]^ The structure contains {Cr_3_}, {Cr_4_}, and {Cr_5_} chains, linking the five Cu^II^ sites in the chain. The structure demonstrates the immense structural flexibility of the {CrF(O_2_C^t^Bu)_2_} building block. This is most clearly seen when Cu^II^ is the divalent metal present, presumably due to the lability of this ion combined with its willingness to adopt distorted coordination geometries.

This ability to control the size of rings through templating ammonium or imidazolium cations allows us to study how the magnetic properties of closed structures vary with size. A particular target is large odd‐numbered rings, which are very rare and which should show spin frustration. Above we mentioned a {Cr_8_Zn} ring **5,** and we have previously reported {Cr_8_Ni},^[^ ^15^ ^]^ {Cr_7_(VO)_2_},^[^ ^15^ ^]^ and {Cr_8_Mn} rings.^[^ ^16^ ^]^ A particular target are homometallic {Cr_9_} rings, and we have described such rings but where one edge is different to the other eight.^[^ ^17^ ^]^ Based on this work, we have developed the synthesis of [CrF(O_2_C^t^Bu)_2_]_9_ **13**.^[^ ^18^ ^]^ The procedure involves adding diisopropylamine to the reaction that gives **2**. This leads to formation of **13**, **2,** and other nine‐metal rings [NH_2_^i^Pr_2_][Cr_9_F_10_(O_2_C^t^Bu)_18_] and [NH_2_^i^Pr_2_][Cr_9_F_11_(O_2_C^t^Bu)_17_],^[^ ^17^ ^]^ which can be separated by chromatography. **13** does not contain diisopropylammonium but does not form if the amine is not included in the reaction.^[^ ^18^ ^]^

**Figure 7:**
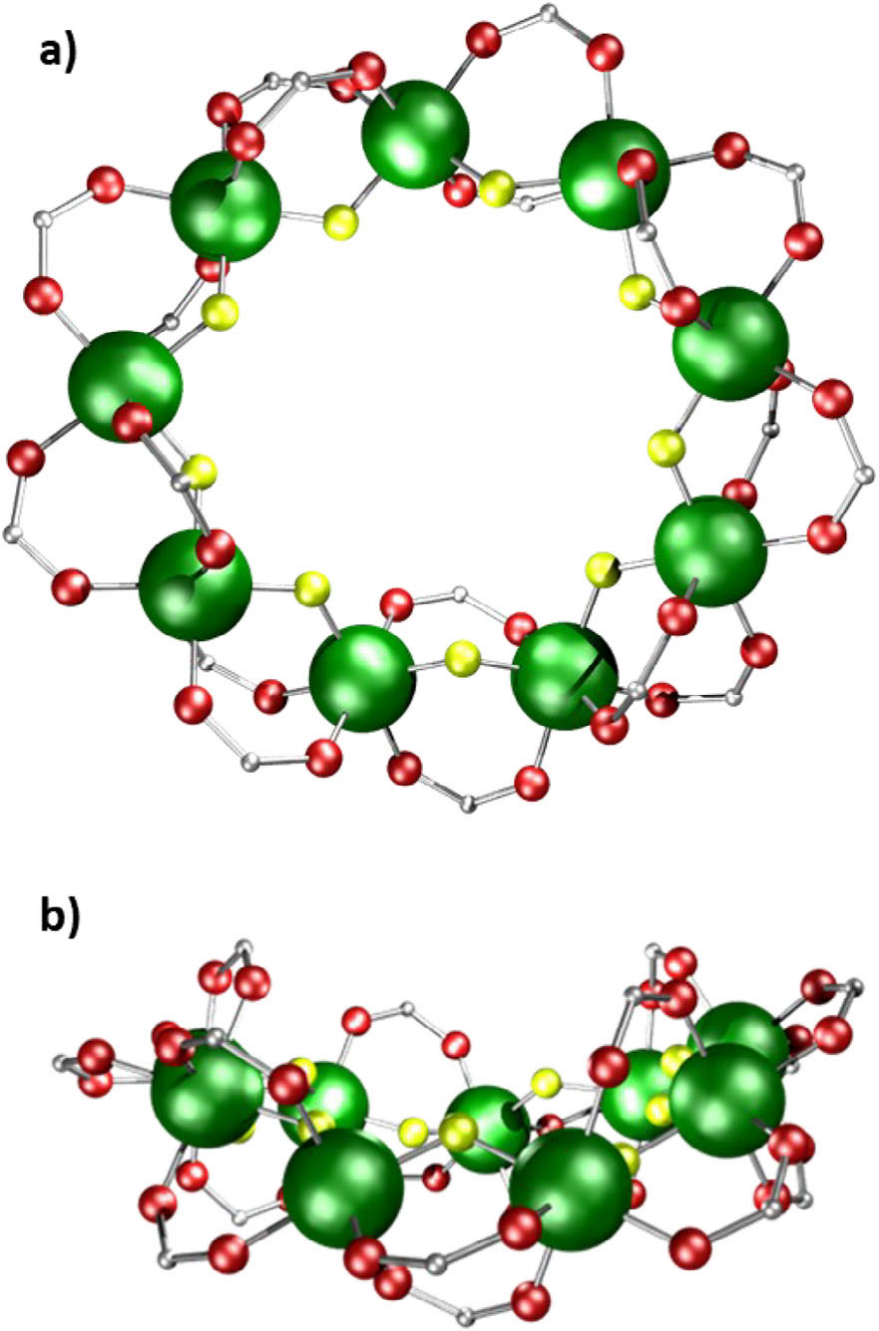
Structures of **13** a) front view, b) side view depicting bowl‐style geometry. Atom colors in Figure 1. Hydrogens and *^t^* Bu groups omitted for clarity.

The structure of **13** contains a bowl‐shaped nine‐metal ring, with each edge bridged by a fluoride and two pivalates (Figure 7). The metric parameters at each Cr^III^ and on each Cr…Cr edge are unchanged between **13** and **2**; the Cr^III^ sites retain a regular octahedral geometry. Physical studies of **13** are discussed in detail in section 4, and they demonstrate that **13** is the only example of a nine‐metal ring that approaches Kahn's definition of spin frustration.^[^ ^19^ ^]^

The {CrF(O_2_C^t^Bu)_2_} fragment is isostructural with, and has the same charge as, a {Ti^IV^O(O_2_CR)_2_} building block, and eightmetal rings were known for this unit, for example [Ti^IV^O(O_2_CR)_2_]_8_.^[^ ^20^ ^]^ This ring is also used as a secondary building unit in metal‐organic frameworks (MOFs).^[^ ^21^, ^22^ ^]^ Therefore, we reasoned we should be able to make equivalent HRMs based on a {Ti^IV^_7_M^III^} ring by analogy with {Cr^III^_7_M^II^}. This is correct, and eight‐, nine‐, and ten‐metal rings can be made by varying the cation.^[^ ^23^ ^]^ The complexes (^n^Pr_2_NH_2_)[Ti_7_MO_8_(O_2_C^t^Bu)_16_] **14** **M** (M ═ Cr^III^, Fe^III^, Al^III^, Ga^III^, or In^III^) can be made from [Ti(^i^PrO)_4_] reacted with pivalic acid in the presence of a trivalent metal source. Changing the template gives (^i^Pr_2_NH_2_)[Ti_8_MO_9_(O_2_C^t^Bu)_18_] **15** **M** (M ═ Fe^III^ or Ga^III^) or the ten‐metal ring (Cy_2_MeNH)[Ti_9_Fe^III^O_10_(O_2_C^t^Bu)_20_] **16**. The **14Ga** ring is a promising diamagnetic host for studying the behavior of the {Cr_7_Ni} rings as qubits.

**Scheme 2:**
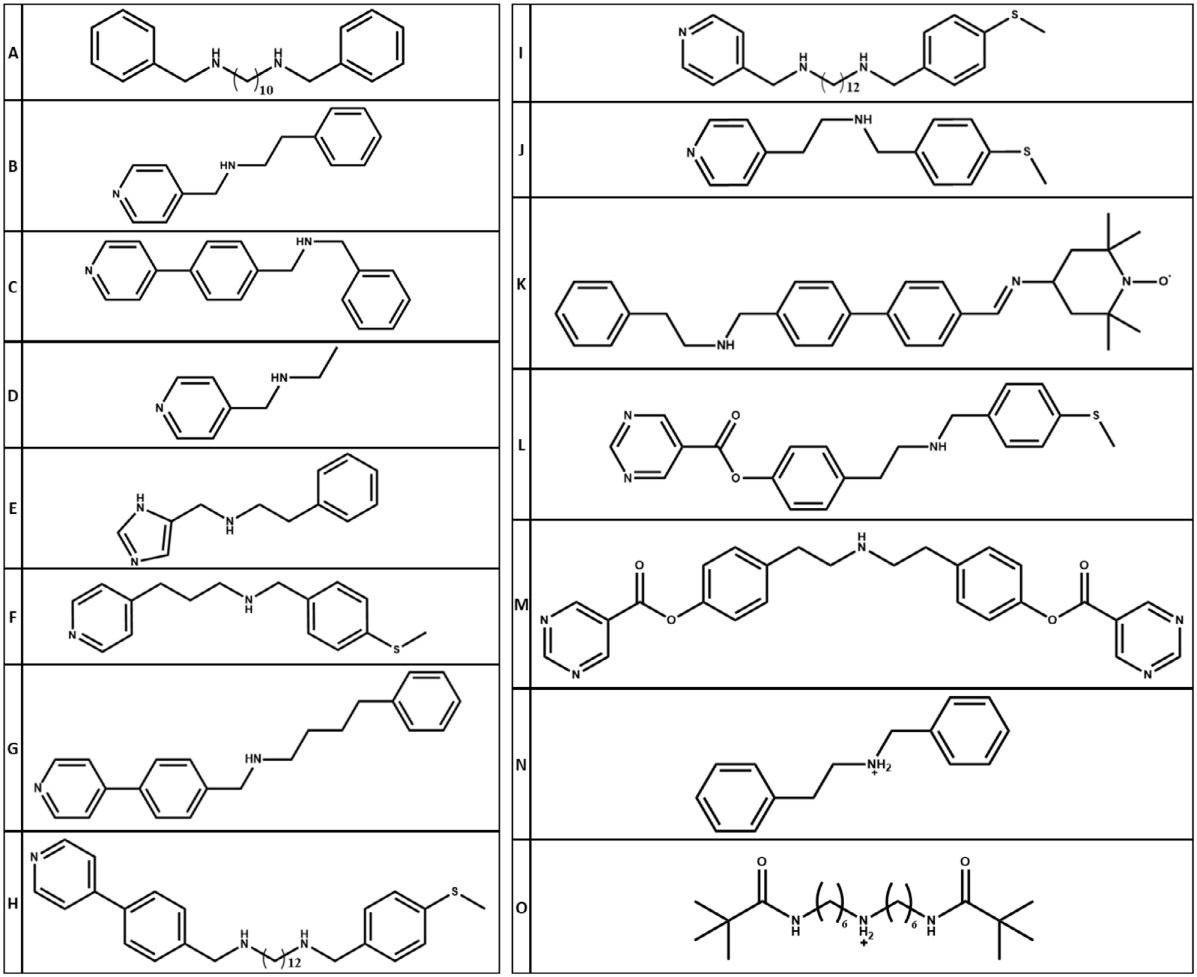
The structures of the threads discussed in this article.

**Figure 8:**
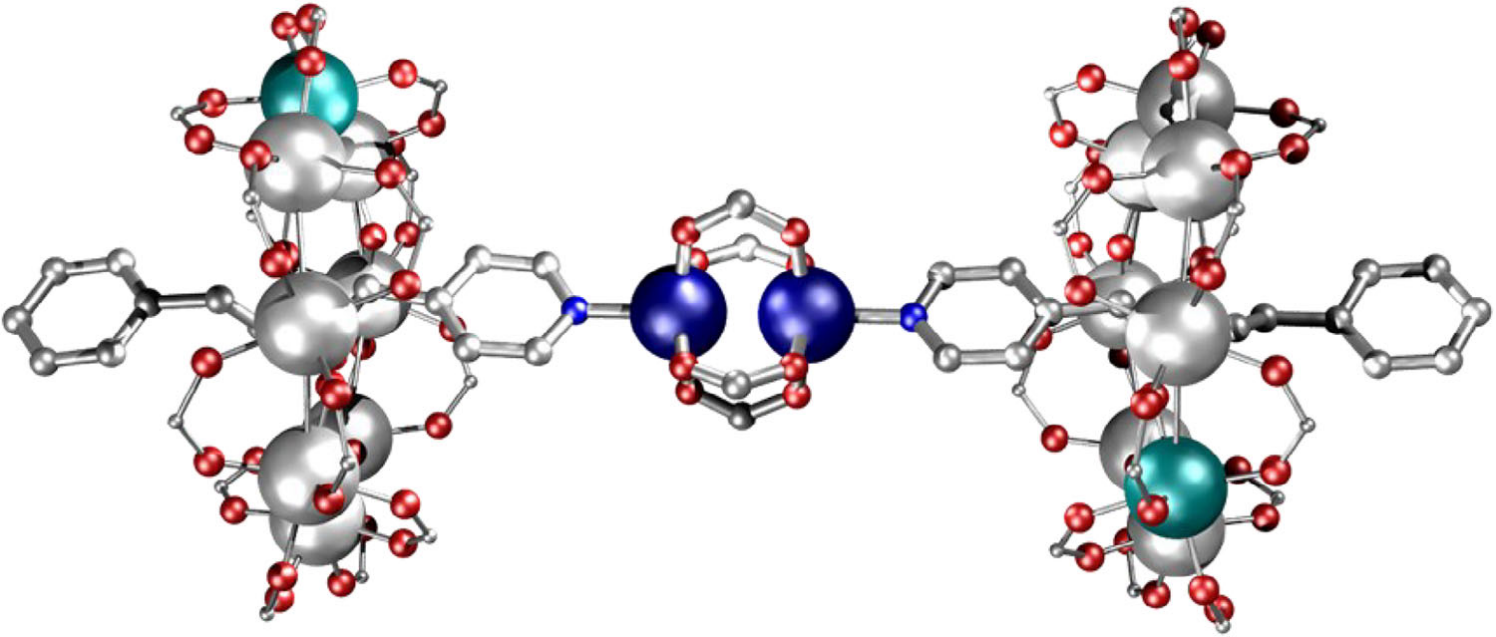
Structures of {(**18Fe**)_2_[Cu(O_2_C^t^Bu)_2_]_2_}. Atom colors in Figure 5, with Ti light grey and Fe teal. Hydrogens and *^t^* Bu groups omitted for clarity.

This premise led to the synthesis of [3]rotaxanes involving {Ti^IV^_7_M^III^} rings.^[^ ^24^ ^]^ Using (PhCH_2_NH)_2_(CH_2_)_10_ **A** as a template (Scheme 2) gives [3]rotaxanes [(**AH_2_**){Ti_7_MO_8_(O_2_C^t^Bu)_16_}_2_] **17** **M** (M ═ Cr^III^, Fe^III^, Ga^III^, or Mn^III^), which feature a C_10_ chain between the two HRMs. If a C_8_ link is used, then only one ring can be grown around the thread, giving [2]rotaxanes. A further [2]rotaxane can be formed terminated with pyridine, that is, (PyCH_2_NHCH_2_CH_2_Ph) **B,** to give [(**B**H){Ti_7_MO_8_(O_2_C^t^Bu)_16_}] **18** **M** (M ═ Fe^III^ or Ga^III^). These [2]rotaxanes can be used as ligands for [Cu(hfac)_2_] (hfac = 1,1,1,6,6,6‐hexafluoroacetylacetonate) or [Cu(O_2_C^t^Bu)_2_]_2_, reacting in 1:1 and 2:1 ratios with these copper complexes, respectively; the latter reaction gives a further [3]rotaxane which features the copper lantern structure between the two {Ti_7_M} rings (Figure 8). The most unusual reaction also involves **A**, where it is possible to grow a {Cr_7_Ni} ring around one ammonium and a {Ti_7_Fe} ring around the second, giving a [3]rotaxane that features two distinct HRMs.^[^ ^24^ ^]^

## 3 Linking Rings

These last results anticipate our next section, which is on linking rings, specifically looking for applications in quantum information processing, but also to examine how much chemistry can be performed on these novel building blocks. The molecules are then acting as qubits, the quantum equivalent of a bit in classical computing, and a key measure of the quality of a single qubit is how long the quantum phase information is stored before being lost. The key aim is to make the phase memory time (also called the coherence time and, occasionally, the decoherence time) as long as possible. A spectroscopic measure is the electron spin transverse relaxation time, which can be measured by EPR spectroscopy. Much work has focused on this topic, with remarkable phase memory times achieved, even at room temperature.^[^ ^25^, ^26^, ^27^, ^28^, ^29^, ^30^ ^]^ If a two‐qubit gate is considered, and this is a vital first step if any useful calculations are to be performed, a further measure is the interaction energy between the qubits. This is inversely proportional to the time to perform the gate, which means there is an upper bound to how large the interaction can be. If the interaction is too large, then the system will not perform as two linked qubits but as a single spin, that is, there would be no one‐qubit operations. There are few studies which show the tuning of the interaction energy between qubits and hence the gate time.

There are three distinct methods for linking rings. The first two are based on coordination chemistry, where we can either make a ring that acts as a Lewis acid or a ring that acts as a Lewis base. The third route uses supramolecular chemistry, as described above, where we functionalize the ammonium cation to enable making the cation‐ring ensemble into a rotaxane or pseudo‐rotaxane. This flexibility leads to a wide range of synthetic chemistry, which also allows us to study the interaction between rings as a function of bridge and distance.

**Figure 9:**
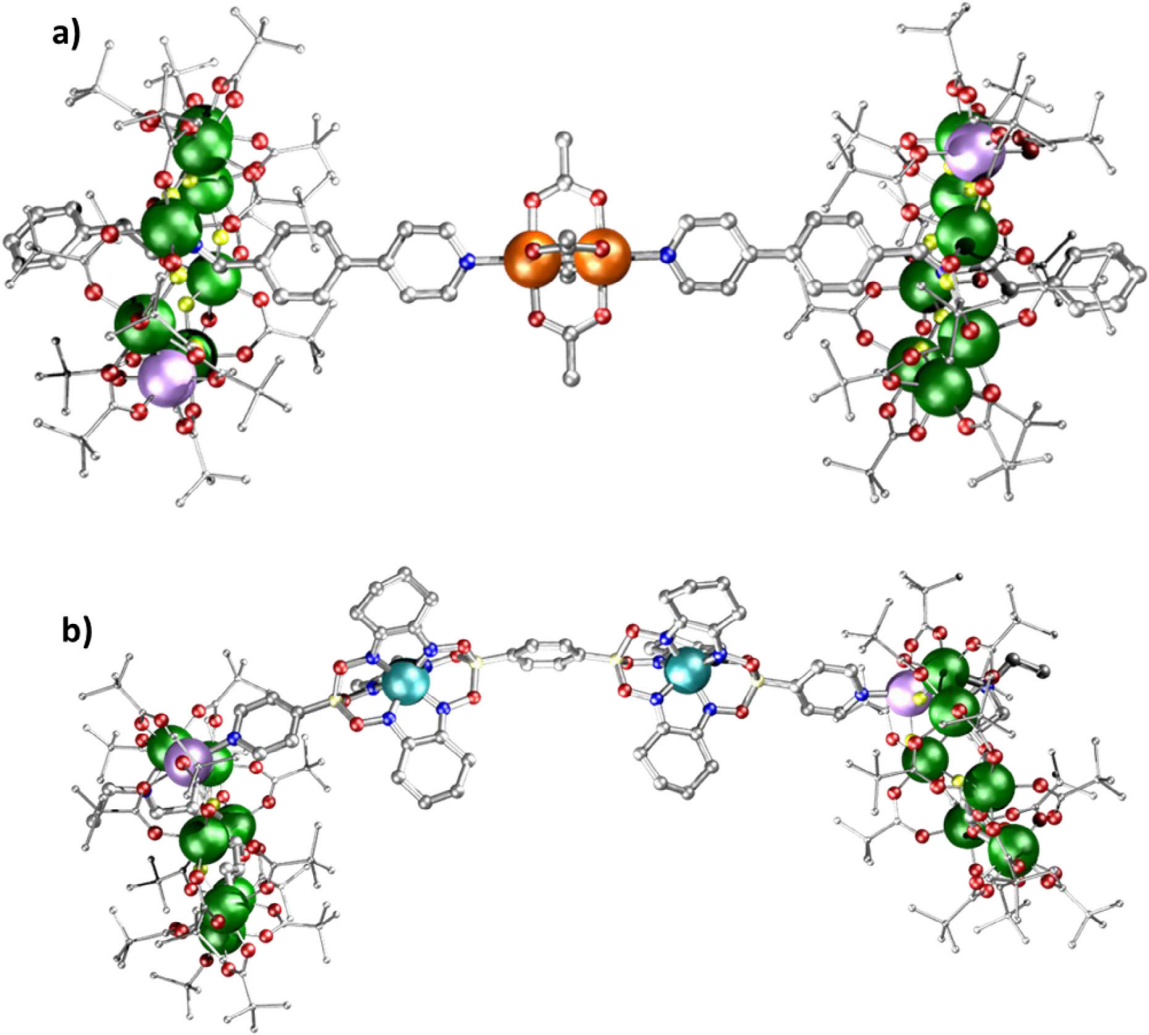
Structures of a) **20** and b) [(**21**)_2 _L] where L = boronic acid‐capped clathrochelate complex. Atom colors in Figure 8, with B; light yellow. Hydrogens omitted for clarity.

The use of rings as Lewis acids and the rotaxane approach was used to produce multiple complexes that allowed us to engineer the interaction energy between the rings. By modifying the carboxylate ligand, it was also possible to tune the phase memory time for the *S* = 1/2 state of the {Cr_7_M} rings. Two distinct families were studied.^[^ ^31^ ^]^ The first contains [3]rotaxanes where two rings are linked by threads containing three *o*‐C_6_H_4_ groups between rings, i.e., [R─CH_2_CH_2_─NH_2_─CH_2_─(C_6_H_4_)_3_─CH_2_─NH_2_─CH_2_CH_2_─R][Cr_7_NiF_8_(O_2_CR’)_16_]_2_ **19** (R = Ph, R’ = ^t^Bu or adamantyl; R = CH_2_CHMe_2_, R’ = ^t^Bu or *d^9^*‐^t^Bu) giving a 16.4 Å distance between ring centroids. A longer link can be achieved using a thread comprising (Ph─CH_2_NH─CH_2_─C_6_H_4_─py) **C** (Scheme 2) linked to [Rh_2_(O_2_CMe)_4_] to give {[**C**H][Cr_7_NiF_8_(O_2_C_t_Bu)_16_]}_2_[Rh_2_(O_2_CMe)_4_] **20** (Figure 9a), giving a 25.0 Å distance between ring centroids.

The second family involves chiral {Cr_7_Ni} rings that are grown about a penta‐deprotonated *N*‐ethyl‐*D*‐glucamine unit (NEtglu)^5−^, [Cr_7_NiF_3_(NEtglu) (O_2_C^t^Bu)_15 _L] **21**, where L is a monodentate ligand that is easily displaced.^[^ ^32^ ^]^ We have previously linked **21** using di‐imines,^[^ ^33^ ^]^ and varied the Ni…Ni distance from ca. 7.0 to 19.9 Å, and we used the boronic acid‐capped clathrochelate complexes developed by Severin and co‐workers^[^ ^34^ ^]^ to extend the distance to as far as 30.7 Å (Figure 9b). By studying **19**, **20,** and dimers of **21,** it was possible to show that the phase memory time can be tuned from 540 to 3240 ns at liquid helium temperatures, and to tune the time for a fixed two‐qubit gate from 80 to 550 ns. This ability to tune two key times separately could be valuable in future.^[^ ^25^, ^31^ ^]^

**Figure 10:**
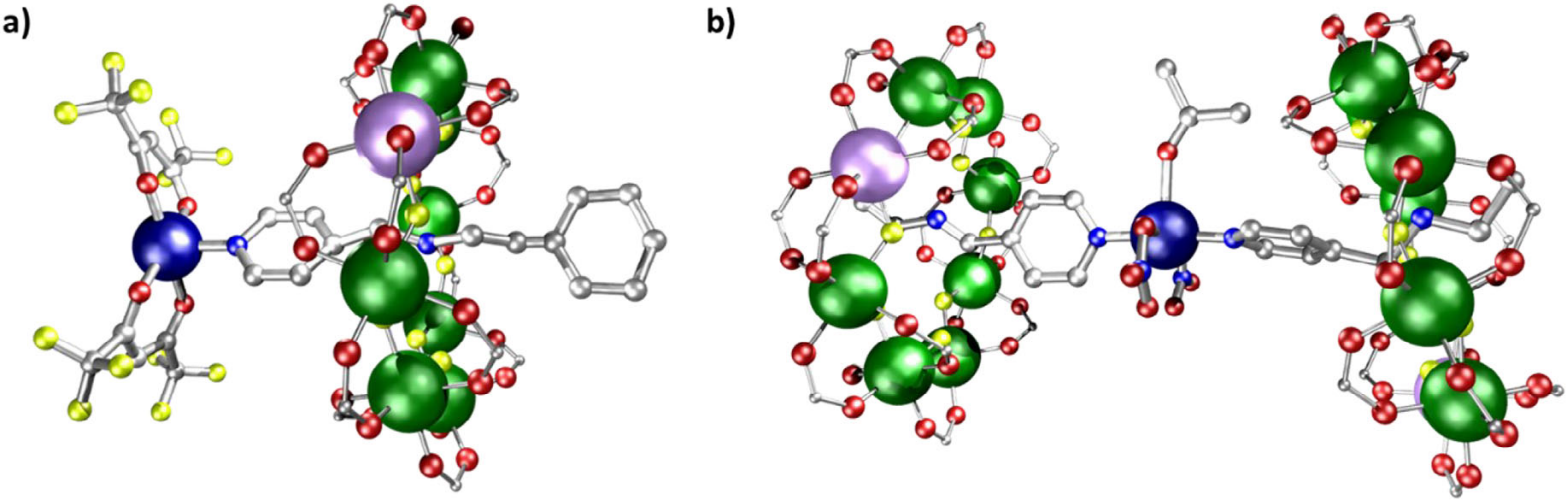
Structures of a) **22** and b) **23**. Atom colors in Figure 5. Hydrogens and *^t^* Bu groups omitted for clarity.

Compounds **19** and **20** are [3]rotaxanes; much larger rotaxanes can also be made. The most straightforward route involves adding a pyridyl at the terminus of a thread within the ring, making a [2]rotaxane (or pseudo‐rotaxane) that can act as a ligand. The distance between the ammonium group, which centers the heterometallic ring, and the pyridyl head group can be easily varied, which allows the interaction energy between the ring and another spin center to be varied. This is illustrated in Table 1, which collects data for the [2]rotaxane bound to [Cu(hfac)_2_].^[^ ^35^, ^36^ ^]^ The structure of these [2]rotaxanes is illustrated by [**B**H][Cr_7_NiF_8_(O_2_C^t^Bu)_16_][Cu(hfac)_2_] **22** (Figure 10a). EPR studies of these compounds allow the exchange interaction to be measured; a measurement that would be impossible by conventional magnetometry as the magnitude of the exchange is so small (they form an AX spin system, see below).

**Table 1:** Control of exchange interactions through choice of threads in [2]rotaxanes.

Thread in [2]rotaxane	Ring centroid…radical^[^ ^a^ ^]^ distance through space [Å]	*J* [cm^−1^]^[^ ^b^ ^]^	Refs.
**B**	7.16(3)	+0.030	[35, 36]
**D**	7.14(3)	+0.026	[36]
**E**	6.30(1)	+0.060	[37]
**F**	9.27(1)	±0.002	[37]
**G**	11.44(10)	<±0.002	[36]
**J**	8.59(8)	±0.010	[36]
**K**	16.83(10)	ca. ±0.000005	[38]
**L**	12.48(1)	<±0.002	[39]
**M**	12.60(1)	<±0.002	[39]

^[a]^ Radical = Cu^2+^ except **K** where it is a nitroxide;

^[b]^ Exchange coupling constants are given in the + *J*ŝ_1_.ŝ_2_ formalism.

**Figure 11:**
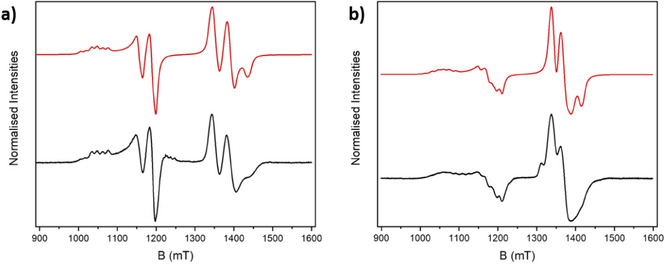
Experimental Q‐band (*ca*. 34 GHz) EPR spectra (black) and simulations (red) for (a) **22**, (b) **23** in 1:1 toluene/CH_2_Cl_2_ solution at 5 K. Simulation parameters: (a) $g_{x,y,z}^{Cr7Ni}$ = 1.780, 1.765, 1.710; $g_{x,y,z}^{Cu}$ = 2.065, 2.045, 2.325; $A_{z}^{Cu}$ = 450 MHz; *J* = +0.032 cm^−1^; (b) $g_{x,y,z}^{Cr7Ni}$ = 1.785, 1.775, 1.715; $g_{x,y,z}^{Cu}$ = 2.050, 2.020, 2.287; $A_{z}^{Cu}$ = 450 MHz; *J* = +0.020 cm^−1^. Data from reference [35].

The only variable parameter in the fit shown in Figure 11 is the exchange interaction, which implies that the spin Hamiltonian parameters associated with the building blocks are retained in the supramolecular structures. [All exchange coupling constants in this review are expressed in the +*J*ŝ_1_.ŝ_2_ formalism; where the original article uses a different Hamiltonian, we have converted the *J* constants to be consistent with this.] As mentioned above, the ability to control and measure the exchange interaction is key to controlling two‐qubit gate times. If [Cu(hfac)_2_] is replaced by hydrated copper nitrate in this chemistry a [3]rotaxane is found,^[^ ^28^ ^]^ where a [Cu(NO_3_)_2_(H_2_O)] complex is bound to two [2]rotaxanes via the pyridyl groups to give [Cu(NO_3_)_2_(MeC(O)Me)]{[**D**H][Cr_7_NiF_8_(O_2_C^t^Bu)_16_]}_2_ **23** (Figure 10b) (**D** is shown in Scheme 2). Here the EPR spectroscopy shows an AX_2_ spin pattern (Figure 11b), that is, coupling of one‐electron spin of type A with two electron spins of type X, where the coupling is much smaller than the difference in resonance frequency. AX and AX_2_ spin systems are often found in NMR, but it is unusual in c.w. EPR to have the difference in the Zeeman energy between the spins greater than the exchange interaction yet for the exchange to be resolved.

This structure, where there are two distinct *S* = 1/2 centers, with one showing a hyperfine interaction to an *I* = 3/2 nucleus, fits with a proposal for performing quantum error corrections (QEC).^[^ ^37^ ^]^ The ability to tune the exchange interaction precisely over several orders of magnitude is important, as it allows us to optimize a molecule for the QEC protocol proposed. By replacing the pyridine with an imidazole group, the largest exchange interaction between the Cu and the Cr_7_Ni rotaxane is achieved, in [Cu(hfac)_2_][**E**H][Cr_7_NiF_8_(O_2_C*^t^* Bu)_16_] **24**. The smallest interaction that is resolvable within the intrinsic linewidth uses a thread with a py(CH_2_)_3_ chain, [Cu(hfac)_2_][**F**H]_2_[Cr_7_NiF_8_(O_2_C*^t^* Bu)_16_]_2_ **25** (**E** and **F** are shown in Scheme 2).

This control of the interaction energy between two spins can be extended because the HRMs can be substituted in two distinct ways.^[^ ^40^ ^]^ We've previously reported substitution of a pivalate on a Cr…Ni edge with an *iso*‐nicotinate to give [^n^Pr_2_NH_2_][Cr_7_NiF_8_(O_2_C^t^Bu)_15_(O_2_C‐py)] **26**;^[^ ^41^ ^]^ coordinating this to a Cu^II^ center leads to {[Cu(NO_3_)_2_(H_2_O)](**26**)_2_} **27,** where the three *s* = 1/2 centers are more strongly coupled, with an exchange interaction of ca. *J* = ‐0.31 cm^−1^. This produces a system where all three possible coupled spins states – an *S* = 3/2 and two *S* = 1/2 states – are occupied at 5 K, but each with distinct *g*‐values.

**Figure 12:**
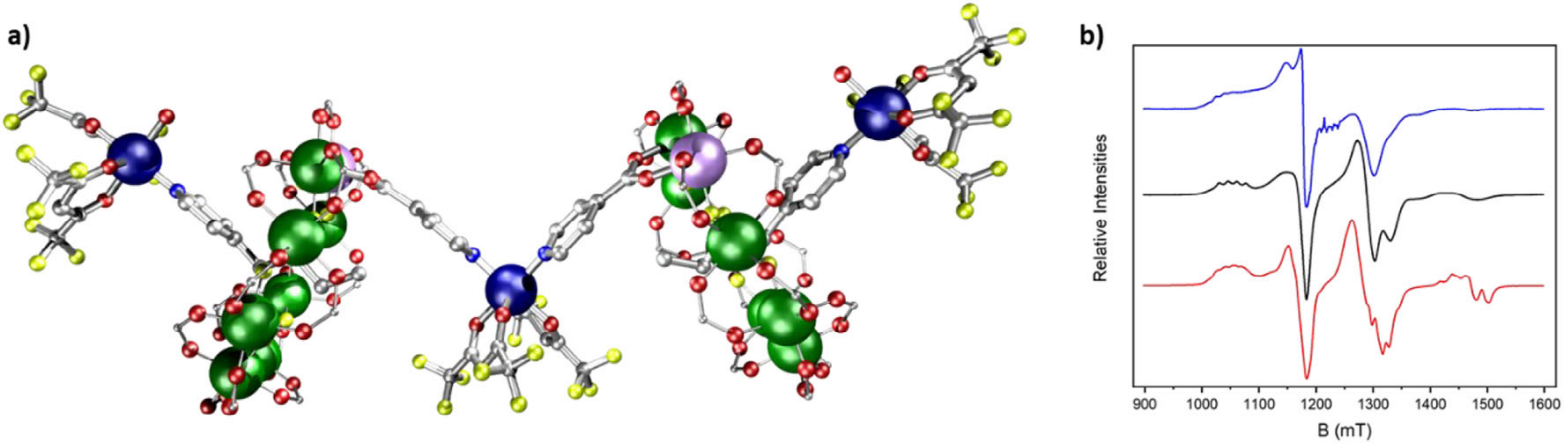
Structure a) **29** and b) Q‐Band EPR (c.a. 34 GHz) for **29**. Experimental solution (blue), powder (black), and calculated spectrum (red). Atom colors as Figure 5. Hydrogens and *^t^* Bu groups omitted for clarity.

By including a thread functionalized with a pyridyl as a guest within the ring, it is possible to make a compound [(**D**H) (Cr_7_NiF_8_(O_2_C^t^Bu)_15_(O_2_C‐Py)] **28** with two distinct pyridyls and which offer two interaction energies, differing by an order of magnitude.^[^ ^40^ ^]^ Mixing this compound with [Cu(hfac)_2_] produces a discrete five‐spin supramolecule [{[Cu(hfac)_2_]**28**}_2_[Cu(hfac)_2_]] **29** (Figure 12a). The distinct interaction energies can be seen by calculating an EPR spectrum based on previous measurements on the two building blocks, i.e., the +0.03 cm^−1^ exchange interaction from the rotaxane **22** and the +0.3 cm^−1^ exchange from **27,** and finding this matches the observed solution and powder EPR spectra of **29** (Figure 12b). The spin Hamiltonian parameters are transferable between the compounds. The combination of interaction energies and coherence times in **29** has led to a proposal that this could be used to model coherence in strongly entangled states, where the central unit, which has the three spins aligned (at least at low enough temperature), could be perturbed by EPR transitions at the peripheral copper sites.

**Figure 13:**
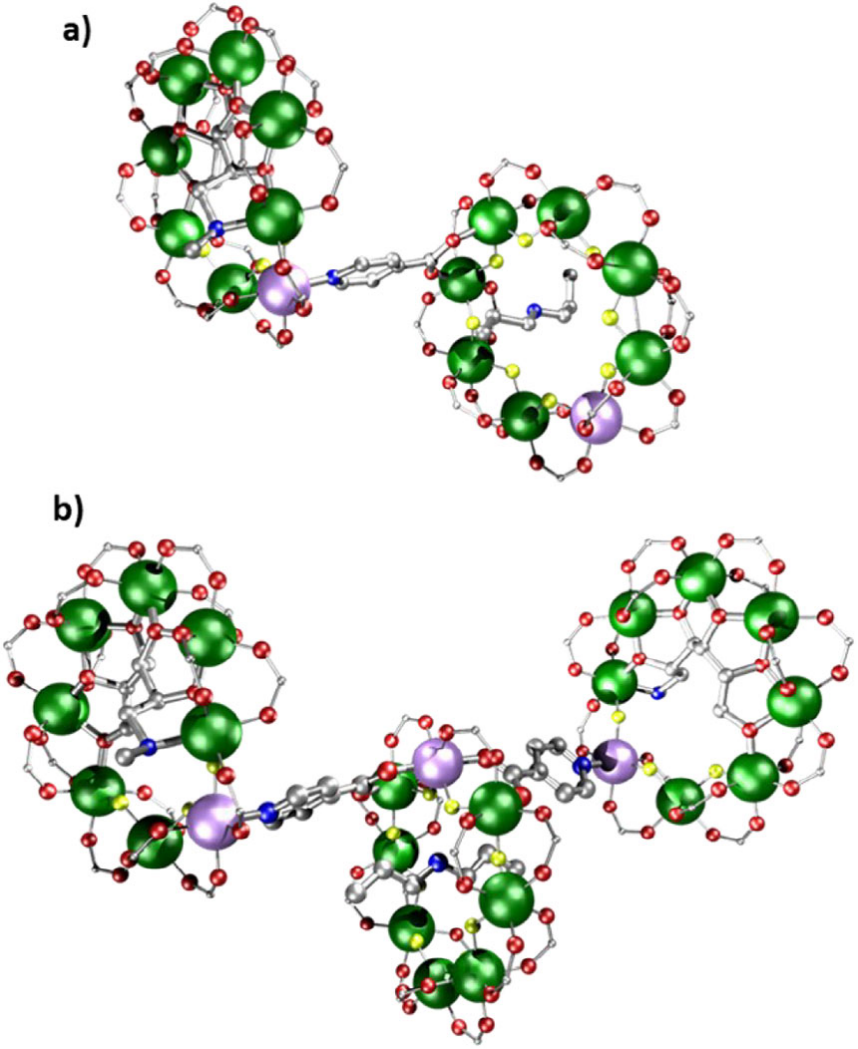
Structure of a) [(**21**) (**26**)] and b) [(**21**)_2_(**30**)]. Atom colors in Figure 1. Hydrogens and *^t^* Bu groups omitted for clarity.

Similar transferability of spin Hamiltonian parameters can be seen by using either **26** or a doubly substituted ring [^n^Pr_2_NH_2_][Cr_7_NiF_8_(O_2_C^t^Bu)_14_(O_2_C‐py)_2_] **30** as ligands for the chiral ring **21**, giving a dimer [(**21**) (**26**)] and trimer [(**21**)_2_(**30**)] (Figure 13a and 13b, respectively).^[^ ^42^ ^]^ The interaction between the ring spins is the same in both, as measured by EPR spectroscopy. By addition of pyridyls at both ends of the thread within a [2]rotaxane, a trimer containing **21** as stoppers at both ends of the [2]rotaxane can also be made. Here the interaction energy is far smaller, as would be expected.

The ability to make rotaxanes where the thread is terminated by a pyridine allows a much wider range of [n]rotaxanes to be made (Table 2).^[^ ^43^ ^]^ Using the thread 4‐phenyl‐*N*‐(4‐(pyridin‐4‐yl)benzylbutan‐1‐amine **G** produces a [2]rotaxane [**G**H][Cr_7_NiF_8_(O_2_C^t^Bu)_16_] **31** (**G** is shown in Scheme 2). This can then be used as a ligand for [Cu(hfac)_2_] to produce a [3]rotaxane [Cu(hfac)_2_(**31**)_2_] **32** or, for an oxo‐centered metal triangle, giving a triangular [4]rotaxane [Fe^III^_2_Co^II^(μ_3_‐O) (O_2_C^t^Bu)_6_(**31**)_3_] **33**. The thread can be extended to include two ammonium stations, for example in *N* ^1^‐(4‐(methylthio)benzyl)‐*N* ^12^‐(4‐(pyridin‐4‐ylmethyl)benzyl)dodecane‐1,12 diamine **H** and in *N* ^1^‐(4‐(methylthio) benzyl)‐*N* ^12^‐(pyridin‐4‐ylmethyl)dodecane‐1,12‐diamine **I** (**H** and **I** are shown in Scheme 2). These form [3]rotaxanes once the {Cr_7_Ni} rings are attached. Coordination of the terminal pyridyls of these threads leads to further rotaxanes, for example the [5]rotaxane [{Cu(O_2_C^t^Bu)_2_}[[**H**H_2_]{Cr_7_NiF_8_(O_2_C^t^Bu)_16_}_2_]_2_] **34** or a [7]rotaxane [{Fe_2_Co(μ_3_‐O)(O_2_C^t^Bu)_6_}{[**I**H_2_]{Cr_7_NiF_8_(O_2_C^t^Bu)_16_}_2_}_3_] **35**.^[^ ^43^ ^]^ The [7]rotaxane did not crystallize but its identity could be confirmed by small‐angle X‐ray scattering (SAXS) in solution.

**Table 2:** Structurally characterized [n]rotaxanes.

[n]	Code	Formula	Shape	Refs.
3	**23**	[Cu(NO_3_)_2_(MeC(O)Me)] {[**D**H][Cr_7_NiF_8_(O_2_C*^t^* Bu)_16_]}_2_	[2]rotaxanes linked via a mononuclear complex	[35]
3	**20**	[Rh_2_(O_2_CMe)_4_]{[**C**H][Cr_7_NiF_8_(O_2_C_t_Bu)_16_]}_2_	[2]rotaxanes linked via a dinuclear complex	[31]
3	**39**	[CrNi_2_F(O_2_C^t^Bu)_6_(**38**)_2_(THF)]	[2]rotaxanes linked via two sites on a triangle	[49]
4	**32**	[Fe^III^_2_Co^II^(m_3_‐O) (O_2_C^t^Bu)_6_(**31**)_3_]	[2]rotaxanes linked via a triangle	[43]
4	**40**	[CrNi_2_F(O_2_C^t^Bu)_6_(**38**)_3_]	[2]rotaxanes linked via a triangle	[49]
5	**34**	[{Cu(O_2_C^t^Bu)_2_}[[**H**H_2_]{Cr_7_NiF_8_(O_2_C^t^Bu)_16_}_2_]_2_]	[3]rotaxanes linked via a dinuclear complex	[43]
7	**35**	[{Fe_2_Co(μ_3_‐O)(O_2_C^t^Bu)_6_}{[**I**H_2_]{Cr_7_NiF_8_(O_2_C^t^Bu)_16_}_2_}_3_]	[3]rotaxanes linked via a triangle	[43]
7	**36**	[Ni_12_(chp)_12_(O_2_CMe)_12_(H_2_O)_6_(**31**)_6_]	Hexagon surrounded by [2]rotaxanes	[45]
13	**37**	[Pd_6_[H**Z**{Cr_7_NiF_8_(O_2_C*^t^* Bu)_16_}]_12_]	Octahedron with [2]rotaxanes on edges	[47]

**Figure 14:**
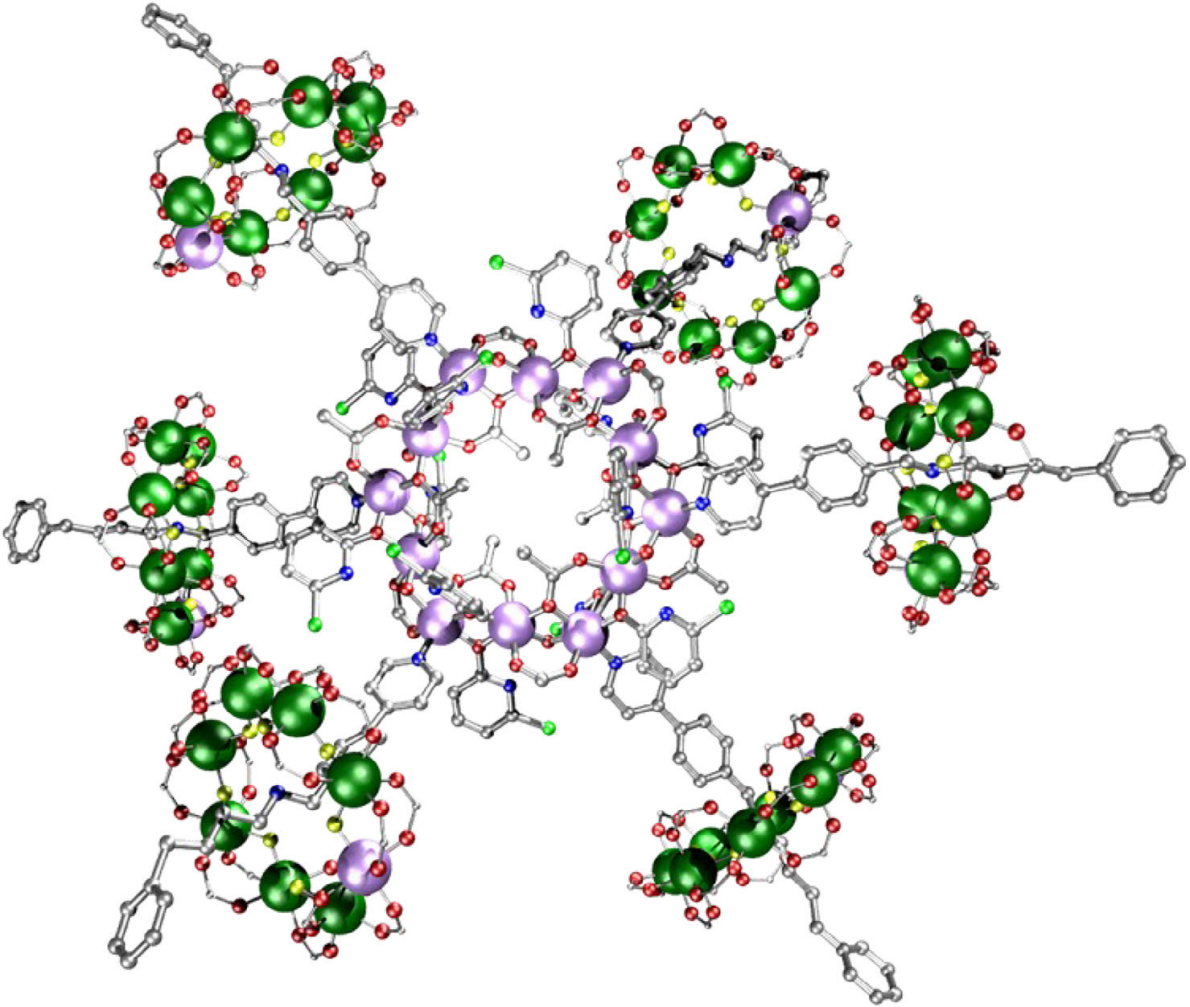
Structure of **36**. Atom colors in Figure 1, with Cl; bright green. Hydrogens and *^t^* Bu groups omitted for clarity.

A more fully characterized family of [7]rotaxanes results from taking similar [2]rotaxanes terminated with pyridyl and reacting them with [Ni_12_(chp)_12_(O_2_CMe)_12_(THF)_6_(H_2_O)_6_] (where Hchp = 6‐chloro‐2‐hydroxypyridine), which is a hexagonal dodecametallic ring.^[^ ^44^ ^]^ The six Ni^II^ sites at the corners of the hexagon are bound to THF molecules, which can be displaced by the [2]rotaxanes, giving [7]rotaxanes, four of which were crystallized; a representative [Ni_12_(chp)_12_(O_2_CMe)_12_(H_2_O)_6_(**31**)_6_] **36** is shown in Figure 14.^[^ ^45^ ^]^ The compounds are related to a previously reported “ring of rings”, which used the same {Ni_12_} core but surrounded it with {Cr_7_Ni} rings functionalized with *iso*‐nicotinate.^[^ ^46^ ^]^ Detailed SAXS studies complimented by atomistic molecular dynamics simulations indicate that the structures are not rigid in solution and do not retain the conformations found in the crystal structure but do retain connectivity in noncoordinating solvents.

The largest crystallographically characterized member of this family is a [13]rotaxane.^[^ ^47^ ^]^ This is made by functionalizing the thread of the [2]rotaxane with a separated bi‐pyridyl ligand; this is based on Fujita's work to make molecular capsules.^[^ ^48^ ^]^ The [2]rotaxane is then reacted with a palladium(II) salt to give [Pd_6_[H**Z**{Cr_7_NiF_8_(O_2_C*^t^* Bu)_16_}]_12_] **37**, where **Z** = 4′‐(2‐(phenethylamino)ethyl)‐[1,1′‐biphenyl]‐4‐yl 3,5‐bis(pyridin‐4‐ylethynyl)benzoate. The [2]rotaxanes bridge Pd…Pd edges of a central polyhedron as expected, but whereas we expected a cuboctahedron, based on the angle between the two bipyridyl groups of each thread, we find a {Pd_6_} octahedron. As there is a [2]rotaxane on each of twelve edges of the octahedron, this is a [13]rotaxane. In addition to these {Cr_7_Ni} rotaxanes, the compound also contains six anionic [Cr_7_NiF_8_(O_2_C^t^Bu)_16_]^−^ empty rings, which are found placed above each Pd^II^ site of the octahedron. They appear to be held in place as they are trapped beneath the carboxylates from the rings within the [13]rotaxane. SAXS measurements demonstrate that these empty rings are held in place in solution.

**Figure 15:**
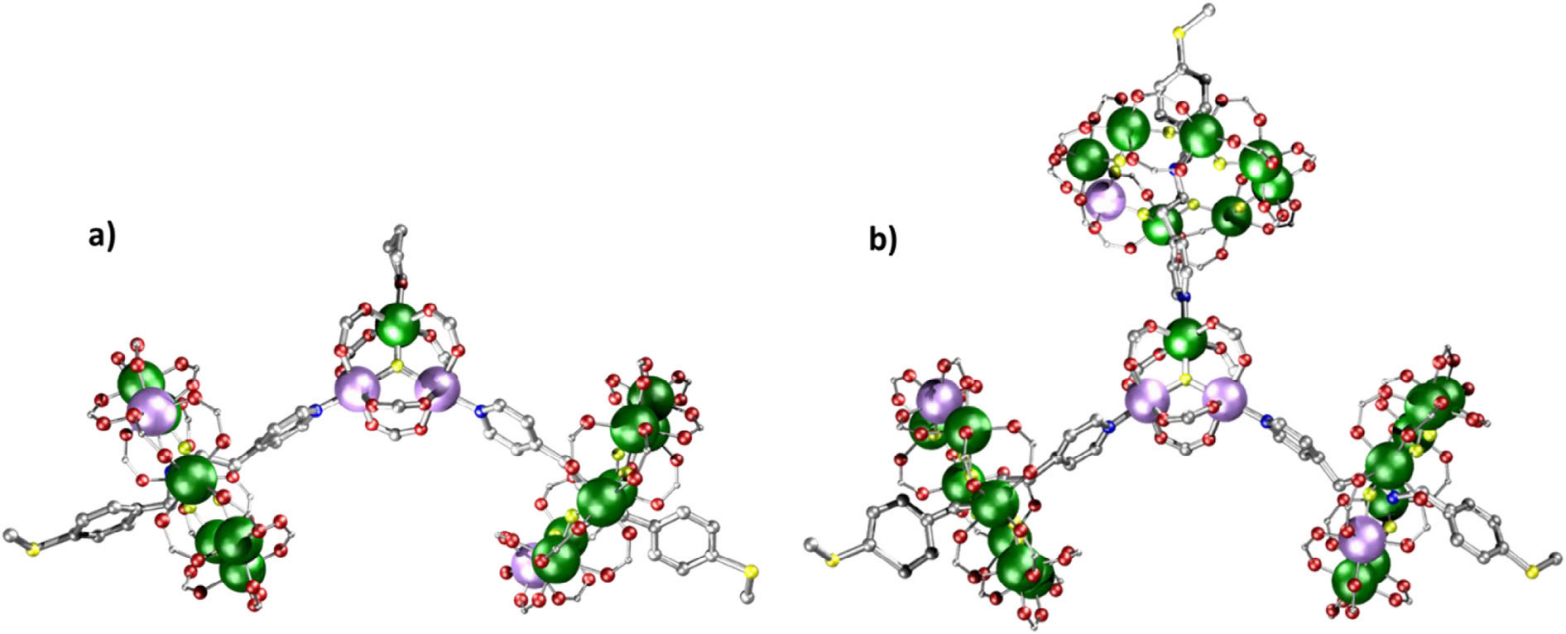
Structure of a) **37** and b) **38**. Atom colors in Figure 1, with S; bright yellow. Hydrogens and *^t^* Bu groups omitted for clarity.

The SAXS measurements on **36** and **37** demonstrate two possible solution behaviors, where in both cases connectivity is retained in solution. In **36** there are many conformers found in solution,^[^ ^45^ ^]^ while in **37** the structure is locked.^[^ ^47^ ^]^ An alternative would be for connectivity to be disrupted, especially as the supramolecule often involves a metal‐ligand bond that could be labile. One example where bond breaking could be an issue concerns binding of [2]rotaxanes to metal triangles. We studied this in two closely related compounds where py‐CH_2_CH_2_NH_2_CH_2_C_6_H_4_SMe **J** was used, forming the [2]rotaxane [**J**H][Cr_7_NiF_8_(O_2_C^t^Bu)_16_] **38**, which was then reacted with a fluoride‐centered triangle [CrNi_2_F(O_2_C^t^Bu)_6_(HO_2_C^t^Bu)_3_] to give a [3]rotaxane [CrNi_2_F(O_2_C^t^Bu)_6_(**38**)_2_(THF)] **39** or a [4]rotaxane [CrNi_2_F(O_2_C^t^Bu)_6_(**38**)_3_] **40** (Figure 15).^[^ ^49^ ^]^ The synthesis of **39** or **40** depends on the mole ratios of [2]rotaxane to triangle (2:1 or 3:1) and on reaction temperature (20 or 40 °C). This seems a remarkable selectivity for coordination chemistry, however both SAXS and double electron‐electron resonance (DEER) spectroscopy^[^ ^50^ ^]^ confirm that **39** and **40** are stable in solution, and there is no evidence of interchange between these two [n]rotaxanes.^[^ ^49^ ^]^ The pyridyl in the thread is substituted in the 4‐position, which requires that the thread lie in the plane of the central {CrNi_2_} triangle, with the {Cr_7_Ni} rings then perpendicular to this plane. This restricts the conformational flexibility of the complex once formed.

If the pyridyl in the threads is substituted in the 3‐position then there is no longer a requirement for the thread to lie in the plane of the {CrNi_2_} triangle, and multiple conformations are possible for any specific connectivity. We have investigated this in four [4]rotaxanes which have either 3‐pyridyl or 3‐pyrimidyl head groups.^[^ ^51^ ^]^ This produces crystal structures where we can find all three [2]rotaxanes on one side of the triangle, almost with a bar‐stool configuration, or one above and two below. DEER spectroscopy, combined with detailed analysis, shows that in solution many conformations are found for these [4]rotaxanes.

An attempt was made to link rings via an Au^I^ center, and here the chemistry shows a different problem.^[^ ^52^ ^]^ This involves substituting in a phosphine‐decorated carboxylate rather than use of a rotaxane, but this illustrates that SAXS and DEER can give seemingly contradictory information, and therefore it fits here in our story. The complex is [^n^Pr_2_NH_2_][Cr_7_NiF_8_(O_2_C^t^Bu)_15_(O_2_C‐C_6_H_4_‐PPh_2_)] **41,** where the phosphine from the ring can bind to Au^I^ centers to give [AuCl(**41**)], [AuCl(**41**)_2_], and [AuCl(**41**)_3_], the latter in very low yield from a reaction intended to give [AuCl(**41**)_2_].^[^ ^45^ ^]^ [AuCl(**41**)] can be reacted with the sodium salt of trithiocyanuric acid to give [(S_3_C_3_N_3_){Au(**41**)_3_}]. SAXS studies of solutions of these compounds show only a single ring is present, i.e., the complexes dissociate to give [AuCl(**41**)] and **41,** and at the temperature of the SAXS experiment (293 K), we only see the monomer. By DEER, measured in frozen solution at 5 K, we see some weak oscillations indicating ring…ring interactions. We believe this indicates that a small amount of [AuCl(**41**)_2_] is present in the frozen solution at low temperature. While these results are disappointing, it does give us confidence that in studies of other systems our SAXS and DEER studies are demonstrating molecules are intact; we know what the observations are when the species dissociate.^[^ ^45^ ^]^

We have focused on using the [2]rotaxanes as ligands for coordination compounds, however, we can develop organic chemistry around this building block. The simplest example is to clip an organic radical onto the thread of the [2]rotaxane to give the thread Ph‐(CH_2_)_2_NHCH_2_(C_6_H_4_)_2_CHN‐TEMPO **K** (Table 1), which forms [**K**H][Cr_7_NiF_8_(O_2_C^t^Bu)_16_] **42** (where TEMPO = 2,2,6,6‐tetramethylpiperidine‐*N*‐oxyl).^[^ ^38^ ^]^ This now includes two distinct spins in the [2]rotaxane, with the organic radical having a longer longitudinal (*T*_1_) relaxation than the {Cr_7_Ni} ring. This allows use of relaxation‐induced dipolar modulation enhancement (RIDME) spectroscopy^[^ ^53^ ^]^ to measure spin…spin distances. An electron spin echo is measured at the nitroxide radical, and the fluctuations of the spin at the {Cr_7_Ni} ring cause oscillations in the dipolar evolution of the radical. The measurements demonstrate that the structure is maintained in solution.

This approach can be taken further with the thread of a [2]rotaxane decorated at one end with a nitroxide, and at the other with a copper(II) porphyrin.^[^ ^48^ ^]^ While this species, containing three distinct spins – Cu^2+^, nitroxide and {Cr_7_Ni} ring – with three distinct *g*‐values could not be crystallized, cw EPR, and RIDME spectroscopy demonstrate that the structure is maintained in solution. RIDME is possible on both the slower relaxing spin centers.^[^ ^54^ ^]^

**Figure 16:**
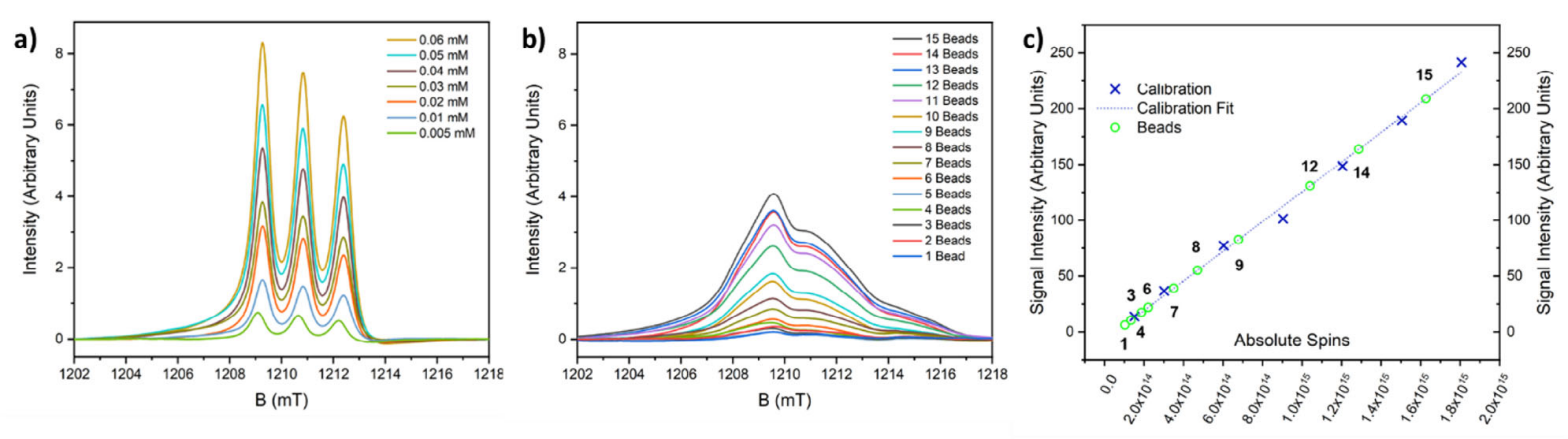
Spin counting measurements for the [10^14^]rotaxanes at room temperature. a) first integral of Q‐band EPR spectra of calibration solutions containing [2]rotaxanes, b) first integral of Q‐band EPR spectra varying number of beads with [2]rotaxanes, and c) spin counting on different numbers of beads, calibrated against a solution of [2]rotaxane. Black figures indicate the number of beads. Measurements for 2, 5, 10, 11, and 13 beads were omitted due to difficulties in obtaining accurate baseline corrections. Data from reference [55].

The ability to include a nitroxide on the thread has also been used to study whether these [2]rotaxanes can be attached to a solid support.^[^ ^55^ ^]^ In collaboration with the Leigh group, we examined using the click reaction to attach [2]rotaxanes to a polystyrene bead; this was mainly a piece of exploratory synthetic chemistry, as we wished to see if the large hybrid inorganic‐organic rotaxane could undergo a simple organic transformation in good yield. The reaction proceeds well for a range of [2]rotaxanes terminated with an alkyne on the thread, reacting with an azide‐decorated bead, however, to determine how many [2]rotaxanes were attached to a bead, we functionalized the other end of the thread with an organic radical. This allows a spin counting experiment to be performed at room temperature, where we can count the number of spins on each bead by cw EPR spectroscopy (Figure 16). This shows that on average there are 10^14^ rings on each bead, making each bead a [10^14^]rotaxane. The measurement matches the calculated number of active sites on the bead remarkably well.^[^ ^55^ ^]^

Finally, by changing the terminal functional group on the thread, we can link the rings into 1‐, 2‐, and 3‐D polymeric compounds, which are MOF materials.^[^ ^39^ ^]^ A [2]rotaxane was prepared with {Cr_7_Ni} on the thread MeS‐C_6_H_4_‐CH_2_‐NH‐CH_2_CH_2_‐C_6_H_4_‐OC(O)‐C_4_H_3_N_2_ **L,** where the ‐C_6_H_3_N_2_ head group is a pyrimidine, to give [**L**H][Cr_7_NiF_8_(O_2_C^t^Bu)_16_] **43**. Reaction with [Cu_2_(O_2_C^t^Bu)_4_] gives the 1D polymer {(**43**)[Cu_2_(O_2_C^t^Bu)_4_]}, where each pyrimidine binds two copper paddlewheel units and the [2]rotaxanes branch out in alternating directions. Functionalizing both ends with pyrimidines in the thread C_4_H_3_N_2_‐C(O)O‐C_6_H_4_‐CH_2_CH_2_‐NH‐CH_2_CH_2_‐C_6_H_4_‐OC(O)‐C_4_H_3_N_2_ **M** gives the [2]rotaxane [**M**H][Cr_7_NiF_8_(O_2_C^t^Bu)_16_] **44**. Reaction with [Cu_2_(O_2_C^t^Bu)_4_] in THF gives the 2D polymer {(**44**)[Cu_2_(O_2_C^t^Bu)_4_]_2_} with a 6,3 net structure with the units of **44** linking between [Cu_2_(O_2_C^t^Bu)_4_](pyrimidine) chains. If instead this reaction is performed in acetone the 3D polymer {(**44**)_2_[Cu_2_(O_2_C^t^Bu)_4_]_3_} is formed, which has a 10,3b net structure.^[^ ^39^ ^]^ The formation of the 2D or 3D structure is determined solely by the solvent, not by stoichiometry.

## 4 Understanding the Chemistry

The ability to make multiple [n]rotaxanes, assembled through either coordination or covalent chemistry, suggests that these HRMs could be used as building blocks more generally in supramolecular chemistry, perhaps occupying the role in molecular machines presently taken by crown ethers. Before that is possible, the physical organic chemistry experiments on stability constants and kinetics that were performed on host‐guest chemistry of crown ethers need to be performed.

Two techniques are useful in this context: NMR spectroscopy, which is possible on these highly paramagnetic species when cobalt(II) is present. The other technique is tandem mass spectrometry, where it is possible to measure the host‐guest binding energy, as long as one or both species is charged.

**Figure 17:**
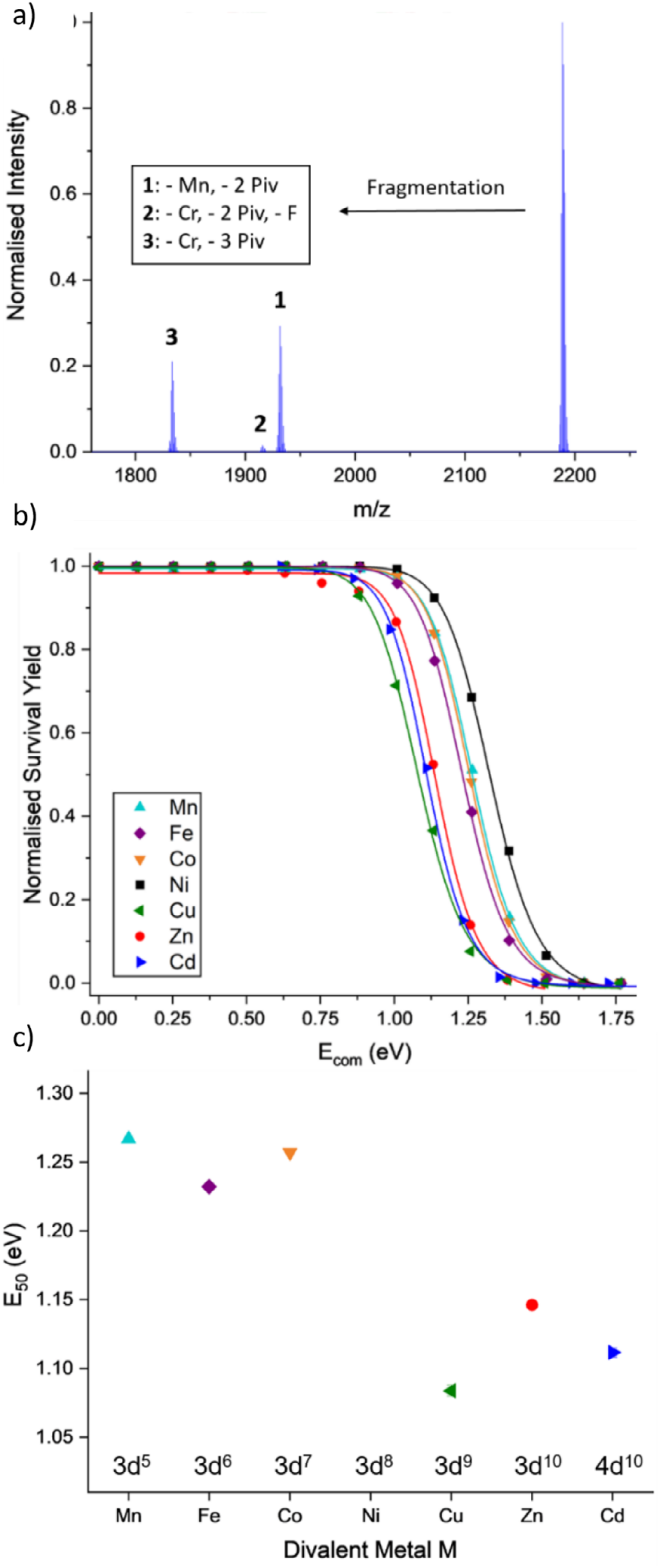
a) Collison‐induced mass spectrometry data from [NH_2_Me_2_][Cr_7_MnF_8_(O_2_C^t^Bu)_16_] at *E*_lab_  =  110 eV, following selection of [Cr_7_MnF_8_(O_2_C^t^Bu)_16_]^−^ (*m/z*  =  2188). b) Normalized survival yield vs *E*_com_ for {Cr_7_M}^−^ rings fitted to a sigmoidal Hill function with M = Mn: cyan, Fe: purple, Co: orange, Ni: black, Cu: green, Zn: red, Cd: blue. c) *E*_50_ values for the dissociation of the anionic rings with respect to M. Figure based on data from reference [56].

Using collision‐induced mass spectrometry (CID), loss of pivalate ligands from [Cr_7_MF_8_(O_2_C^t^Bu)_16_]^‐^ (M = Mn^II^, Fe^II^, Co^II^, Ni^II^, Cu^II^, Zn^II^, or Cd^II^) was studied; the cation used in these experiments was Me_2_NH_2_^+^ which leaves easily, allowing the anion to be studied.^[^ ^56^ ^]^ The anionic ring was collision activated and the energy at which 50% of the original anion disappears (*E*_50_) used as a measure of stability. The major fragment seen is [Cr_7_F_8_(O_2_C^t^Bu)_14_]^−^, i.e., loss of the divalent metal and two pivalates (Figure 17). The next most common fragment is [Cr_6_MF_8_(O_2_C^t^Bu)_13_]^‐^, i.e., loss of a Cr^III^ and three pivalates. The energy to dissociate the anion is largest for M = Ni^II^ and smallest for Cu^II^, presumably reflecting the combination of size and crystal field stabilization energy in the former case and the Jahn‐Teller distortion in the latter. Ion mobility mass spectrometry indicates that [Cr_7_F_8_(O_2_C^t^Bu)_14_]^‐^ predominantly exists as a single acyclic conformer, but that two conformers are seen for [Cr_6_MF_8_(O_2_C^t^Bu)_13_]^‐^ one cyclic and one acyclic.

Using a large ammonium cation that cannot leave allows study of the host‐guest complex. The two ammoniums studied were **N**, [PhCH_2_CH_2_NH_2_CH_2_Ph]^+^ and **O**, [NH_2_{(CH_2_)_6_‐NH(O)^t^Bu}_2_]^+^.^[^ ^56^ ^]^ As the [2]rotaxane is neutral, we study cation forms which are either protonated or sodiated. When studied by CID the sodiated [2]rotaxane preferentially loses a single pivalate and the thread cation, and the *E*_50_ values follow a similar trend to those for decomposition of the anionic ring. This trend also matches the trend in the water exchange reactions for [M(H_2_O)_6_]^2+^. The protonated [2]rotaxane shows a different dissociation path and trend; the *E*_50_ values are much smaller than for the other two collisions studied, and the main fragment involves loss of HO_2_C^t^Bu to give {[**N**][Cr_7_MF_8_(O_2_C^t^Bu)_15_]}^+^ as the main fragment. The trend now follows the Irving‐Williams series with the [2]rotaxane with M = Cu^II^ the most stable. Ion mobility mass spectrometry shows that all these fragment ions, which all contain a {Cr_7_M} core, are all cyclic. This difference between sodiated and protonated [2]rotaxanes led us to consider the adducts with potassium and cesium;^[^ ^57^ ^]^ perhaps predictably they follow the same trends as the sodiated [2]rotaxanes.

Two major conclusions come from this work; first, coordination concepts such as the Irving‐Williams series and influence of Jahn‐Teller distortions are directly applicable to these complex heterometallic rotaxanes. Second, there was the surprising observation of cyclic heptametallic complexes [Cr_6_MF_8_(O_2_C^t^Bu)_13_]^‐^; we have only made one heptametallic ring to this point [Cr_6_CeF_7_(O_2_C^t^Bu)_14_(THF)_2_] **45**, which was isolated in very low yield.^[^ ^58^ ^]^ There are only two further heptametallic rings reported,^[^ ^59^, ^60^ ^]^ therefore, this observation was surprising.

The observation led us to examine related but more complicated species.^[^ ^61^ ^]^ These were [^n^Pr_2_NH_2_]_2_[Cr_10_Cu_2_F_14_(O_2_C^t^Bu)_22_] **46**,^[^ ^62^ ^]^ [^i^Pr_2_NH_2_]_2_[Cr_12_Cu_2_F_16_(O_2_C^t^Bu)_24_] **47** ^[^ ^63^ ^]^ and [^n^Pr_2_NH_2_]_2_[Cr_12_Gd_4_F_21_(O_2_C^t^Bu)_29_] **48**.^[^ ^58^ ^]^ The **46** and **47** complexes are “hourglasses” with the ammonium cations within the two cavities; **48** contains a {Gd_4_} tetrahedron coordinated by two {Cr_6_} horseshoes. Both **47** and **48** therefore contain {Cr_6_} chains. CID studies of **46** and **47** focused on the sodiated cations, and in both cases show significant fragments to heptametallic species; with **47** this has a {Cr_6_Cu} core, which is half of the original complex. With **46** the fragment has a {Cr_5_Cu_2_} core. Ion mobility studies suggest both the {Cr_6_Cu} and {Cr_5_Cu_2_} fragments are cyclic. CID of **48** shows formation of a cyclic fragment with a {Cr_6_Gd_2_} core if the collision energy is 210 eV. This is half of the original molecule and related to an existing structure. With a collision energy of 240 eV we generate a cyclic {Cr_5_Gd_2_} fragment, which is another unexpected heptametallic species. DFT calculations confirm these heptametallic species are stable.^[^ ^61^ ^]^

These studies demonstrate the potential of advanced mass spectrometry in metallosupramolecular chemistry; the techniques used are common in biological chemistry but largely unused in synthetic chemistry. These exploratory studies have already generated synthetic targets, in heptametallic rings we would never have thought to target.

The NMR studies of a family of [R_2_NH_2_][Cr_7_Co^II^F_8_(O_2_CCH_2_^t^Bu)_16_] (R ═ linear alkyls from methyl to hexyl) allowed understanding of the binding energies of these cations, and an understanding of the mechanism for their substitution.^[^ ^64^ ^]^ The carboxylate was changed from pivalate to 2,2‐dimethylbutanoate to improve solubility and optimize the NMR experiments. The NMR spectra are spread over a wide chemical shift range, with the protons from the ammonium cations shifted to negative ppm; the N‐H protons are the most shifted and found around −80 ppm at room temperature. The Me groups from the carboxylates fall in the range of −5 to +10 ppm, while the CH_2_ protons from the carboxylate show large positive chemical shifts to between +15 and +50 ppm. In the absence of a competing amine, no signal is observed from the free ammonium, hence, there is no measurable dissociation in solution: this is in contrast to crown ethers, where the equilibria can be monitored.^[^ ^65^ ^]^

**Figure 18:**
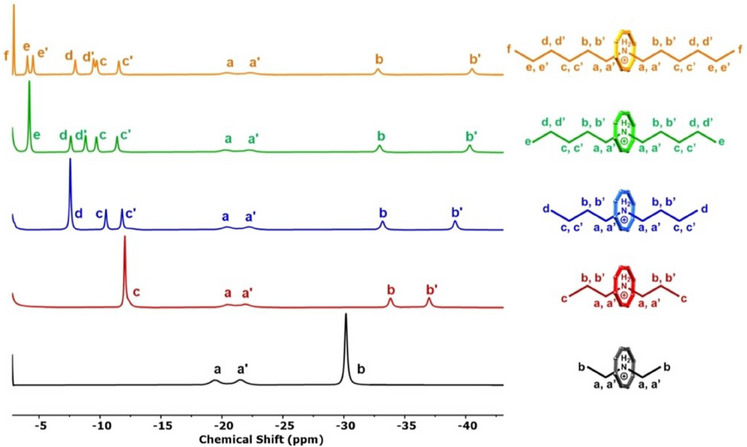
^1^H NMR (500 MHz, 298 K, 30 mM) baseline corrected spectrum (−2.5 to − 45 ppm) of **Et_2_NH_2_** (black), **Pr_2_NH_2_** (red), **Bu_2_NH_2_** (blue), **Pe_2_NH_2_** (green), and **Hx_2_NH_2_** (orange) in toluene‐*d*_8_. Assigned structures of each molecule have been included for clarity. Data from reference [64].

As the ammonium region is isolated and well resolved, the equilibrium constants and kinetics of displacing one dialkylammonium with another can be studied (Figure 18). Both the relative stabilities of different host‐guest complexes and the kinetics are solvent dependent; exchange reactions are slower in *d*‐toluene than *d*‐acetone, indicating a polar transition state, which we assume to be loss of the original dialkylammonium before the second ammonium binds.

The [Et_2_NH_2_]^+^ cation binds most strongly, with the binding similar for the longer alkyl chains.^[^ ^64^ ^]^ The work was extended to primary ammonium cations, which were found to bind much more weakly than secondary ammoniums; this reverses the trend to that found for crown ethers, where the additional H‐bond possible for a primary ammonium leads to greater stability for the host‐guest complex.^[^ ^65^ ^]^ We also find that diallylammonium binds much more weakly than any other dialkylammonium, again in contrast to crown ethers, where there is no strong preference. CID‐MS studies were used to confirm these results, and the match between the trend stability constants in *d*‐acetone and the trend in *E*_50_ values from CID is excellent.

The increased stability of binding of [Et_2_NH_2_]^+^ over other ammonium cations suggests a role for the protons on the carbon chain, and we established a linear correlation between the *E*_50_ values and hydrogens at various positions in the carbon chains. We find protons on the α‐carbon are most important, then β‐protons. This explains why unsaturated side chains such as allyl lead to less firmly bound guests. It also allows us to predict the binding of asymmetric ammoniums, e.g., [^n^PrMeNH_2_]^+^.^[^ ^64^ ^]^

The Gibbs free energy of association for the {Cr_7_Co^II^} ring is between ‐12 and ‐34 kJ mol^−1^, and this is similar to the association energies found for dibenzo[24]crown‐8.^[^ ^66^ ^]^ These NMR and CID studies establish that the {Cr_7_M} rings could be used as components of molecular machines, with similar binding constants to established organic rings.

## 5 Studies of the Physics of Rings and Rotaxanes

The simplicity of the cyclic structures of the original homometallic paramagnetic rings initially attracted interest from physicists as finite models for 1D chains. However, it quickly became apparent that the physics were interesting in their own right; a famous early example was the staircase magnetization behavior observed in an {Fe^III^_10_} ring.^[^ ^67^ ^]^ Earlier physics studies on the HMRs have been reviewed elsewhere^[^ ^6^ ^]^ and these studies included: characterization of internal magnetic structure by magnetometry, INS, and EPR; direct observation of avoided level crossings and fluctuations in the total spin at such crossings, by INS and torque magnetometry; observation of spin‐spin correlations by (4D‐)INS; internal spin distribution studied by single crystal NMR and polarized neutron diffraction; the transferability of spin Hamiltonian parameters between related species and the limits of the giant spin approximation; electron spin dynamics by pulsed EPR; measurement of weak molecule…molecule interactions within supramolecular structures by cw and pulsed EPR. Recent developments in the last topic, For example measuring conformational flexibility of the supramolecules in solution by pulsed EPR (and other techniques) and developing predictable molecule…molecule interactions (measured by EPR), are described above. In this section we focus on other studies, namely: (i) spin frustration effects, (ii) the effect of orbitally degenerate ions on cluster anisotropy, (iii) probing entanglement between molecules by 4D‐INS, (iv) electric field control of molecular spins, and (v) using molecules as a probe of topological insulator behavior.

### 5.1 Spin Frustration Effects

Complex **13**, [CrF(O_2_C^t^Bu)_2_]_9_,^[^ ^18^ ^]^ is a very rare example of a regular homo‐metallic ring with an odd number of metal ions. This makes it an attractive candidate to study spin frustration. In the definition given by Kahn,^[^ ^19^ ^]^ spin frustration arises when there are competing antiferromagnetic interactions that lead to a degenerate ground state. This should arise in an odd‐numbered, odd‐electron ring with equal antiferromagnetic internal couplings about the ring. In an even‐membered ring, the ground state can be simply described as a “spin up ‐ spin down” structure, alternating about the ring, giving *S* = 0. In an odd‐membered ring this is not possible. If the individual spins are half‐integer the ground state in principle is a degenerate pair of *S* = ½ states. However, this is actually very rare because even in the simplest systems (a triangle of spins), the degeneracy tends to be broken by low‐symmetry distortions (a form of the Jahn‐Teller principle).

**Figure 19:**
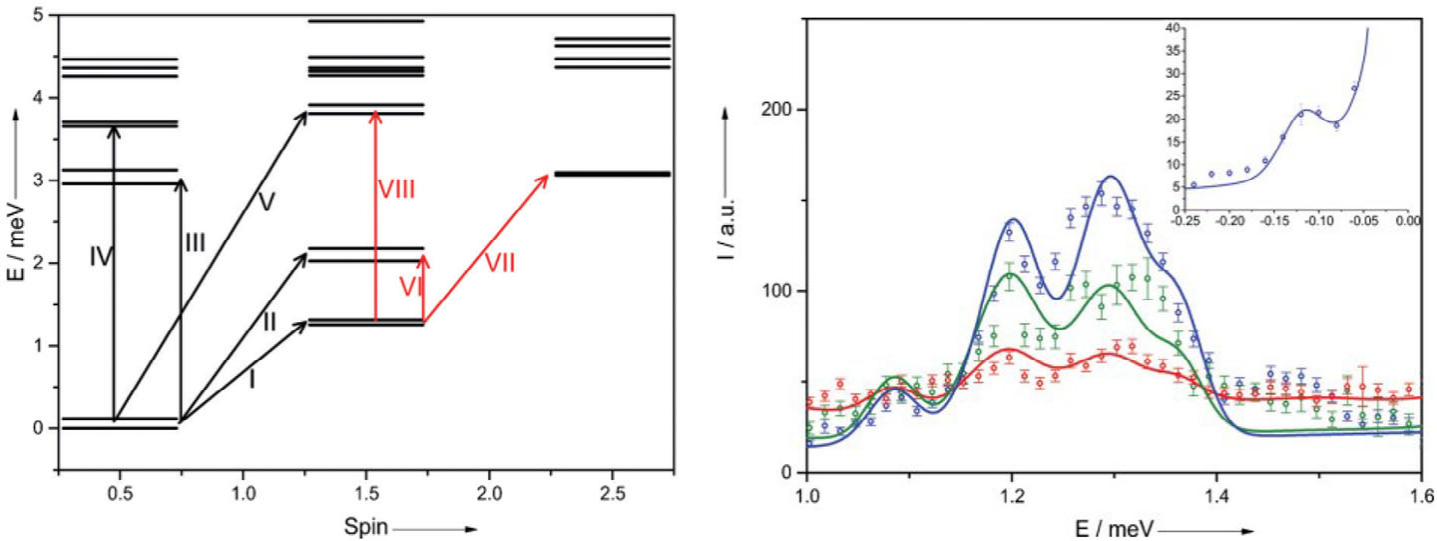
Left: low‐lying level structure of the regular {Cr_9_} complex **13**. Right: INS [2.5 meV neutrons, at 1.5 (blue), 7 (green), and 15 K (red)]. The peaks centered at ca. 1.3 meV correspond to transition I in the left‐hand panel. Inset: High‐resolution INS (1.24 meV neutrons) showing transition between the near degenerate (0.1 meV) *S* = 1/2 ground states. Figure adapted from reference [18].

Magnetic data for **13** could be modeled with a single unique AF exchange interaction of *J* = +10.9 cm^−1^ (+1.35 meV); this should lead to a perfectly frustrated system.^[^ ^18^ ^]^ This was probed by INS, which can measure transitions between spin states directly, as a more sensitive test. Indeed, with 2.5 meV incident neutron energy (corresponding to 58 εeV FWHM resolution at the elastic peak), the INS data were reproducible with a C_9_ symmetric spin Hamiltonian, with the lowest energy peaks observed (at 1.3 meV, or 10.5 cm^−1^) corresponding to excitations from the degenerate pair of *S* = ½ ground states to the *S* = 3/2 first excited state (Figure 19). However, higher resolution measurements (1.24 meV neutrons; 22 εeV FWHM resolution) reveal a 0.1 meV (0.8 cm^−1^) transition between the *S* = ½ states. This data [including the momentum transfer (***Q***) dependence] could be modeled with one edge of the enneagon being slightly different (*J*’ = +12.7 cm^−1^; +1.58 meV) to the other eight (*J* = +10.6 cm^−1^; +1.32 meV). This symmetry breaking is much smaller than in the previously studied irregular {Cr_9_} rings, and is remarkably small given the size of the structure.^[^ ^18^ ^]^ For comparison, the gap between the spin doublets in [NH_2_^i^Pr_2_][Cr_9_F_11_(O_2_C^t^Bu)_17_] is 5.0 cm^−1^, while [NH_2_^i^Pr_2_][Cr_9_F_10_(O_2_C^t^Bu)_18_] actually has an *S* = 3/2 ground state due to one very weak *J* coupling.^[^ ^17^ ^]^ In a separate paper, the consequences for the very near degeneracy on the electron spin dynamics in **13** were probed by variable field and temperature proton spin‐lattice relaxation (1/*T*_1_) measurements by solid‐state ^1^H NMR.^[^ ^68^ ^]^ Experimental data were modeled by a microscopic Hamiltonian including magnetoelastic couplings, allowing interpretation in terms of the phonon‐induced decay of molecular magnetization. Below 3 K, the relaxation dynamics are found to be dominated by a single rate; this can be reproduced from a C_9_ symmetric (i.e., frustrated) model, where the relaxation rate is found to be doubly degenerate, in contrast to previous studies on the even‐numbered ring {Cr_8_}. Models with significant symmetry breaking (even as low as *J*’ = 1.1 *J*) predict relaxation dynamics dominated by two distinct rates, which would then be characteristic of competing, but not frustrated, AF interactions.

An example of an odd‐numbered, odd‐electron HMR with competing AF interactions, but not frustrated, is given by [NH_2_^i^Pr_2_][Cr_8_MnF_9_(O_2_C^t^Bu)_18_] **49**.^[^ ^16^ ^]^ Magnetic data can be modeled with two unique exchange interactions, *J*_CrCr_ = +10.6 and *J*_CrMn_ = +10.3 cm^−1^: the similar AF interactions give an *S* = ½ ground state with *S* = 3/2 and 1/2 first and second excited states at *ca*. 3.2 and 10.5 cm^−1^, respectively, (observed by INS; these data require one subtly different *J*’_CrCr_). The degeneracy breaking here is due to the presence of the Mn^II^, *s* = 5/2 heterospin rather than differences in the *J* couplings. Using these parameters, the strongest nearest‐neighbor spin‐pair correlations are found for the Cr…Mn…Cr fragment due to the larger spin moment of Mn^II^. This unit is then well described as having anti‐parallel spins, where the weaker Cr…Cr correlations can be visualized as noncollinearity of neighboring spins (with the Cr spins on the opposite edge to Mn being near‐perpendicular to that of Mn). The ground state of **49** as a function of an applied magnetic field was also studied by muon spin relaxation (εSR) measurements. In this experiment, spin‐polarized muons are implanted into the sample and emit positrons on their decay. The preferential direction of the emission (quantified by an asymmetry parameter) is related to the muon spin polarization and this can be followed as a function of time. If the field experienced by the implanted muon fluctuates in time, this will result in depolarization. Such effects are enhanced at applied magnetic fields that induce a level crossing between the different total spin states. εSR measurements in variable field and at 100 mK revealed a dip in the asymmetry (and a minimum in the relaxation rate) at around 3.7 T, which is in excellent agreement with the crossing of the *M* = −3/2 sublevel of the lowest *S* = 3/2 state with the M = −1/2 sublevel of the ground state predicted by the INS‐determined *J*‐values. The resonance widths allowed estimation of a crossing gap of a few tens of εeV, and this is consistent with an avoided crossing.^[^ ^16^ ^]^

### 5.2 The Effect of Orbitally Degenerate Ions on Cluster Anisotropy

Metal ions with a large magnetic anisotropy due to unquenched orbital angular momentum, coupled with structural anisotropy, are of interest in several areas of application. However, how the anisotropy of such ions influences that of an exchange‐coupled cluster is poorly understood. Given the wealth of information on the electronic structure of the {Cr_7_M} family, including all the single‐ion and exchange terms involving the Cr^III^ ions, the {Cr_7_Co} analogue seemed an ideal candidate to probe such effects. Such a study was performed on [NH_2_Me_2_][Cr_7_CoF_8_(O_2_C^t^Bu)_16_].^[^ ^69^ ^]^ The Co^II^ ion in {Cr_7_Co} has a large magnetic anisotropy due to its ^4^T_1g_ ground term and the *cis*‐{O_4_F_2_} coordination sphere. Spin‐orbit coupling results in a well‐isolated (by *ca*. 180 K) Kramers doublet, which can be treated as an effective spin *s* = 1/2 for modelling of low‐temperature magnetic data. In this model, the ground state of {Cr_7_Co} is *S* = 1 due to the mismatch with the *s* = 3/2 Cr^III^ spins.

INS measurements, including exploitation of the single‐crystal 4D‐INS experiment first developed to study spin dynamics in the parent {Cr_8_} system,^[^ ^70^ ^]^ revealed lowest energy excitations (at 1.5 K in nil applied field) at 0.1 and 0.5 meV (0.8 and 4.0 cm^−1^, respectively). The origin of these peaks was determined unambiguously to be intra‐ground state by their magnetic field dependence. The entire data set could be modelled with the isotropic part of the Cr…Co interactions as *J*_CrCo_ = +13 cm^−1^, and the diagonal values of the anisotropic exchange interaction matrix as *D*_xx,yy,zz_ = −2, +9, −7 cm^−1^ (where z is the Cr…Co vector and x is normal to the {Cr_7_Co} plane), which are in good agreement with values derived from ab initio calculations. The modeling used Co^II^ effective *g*‐values experimentally measured from the isostructural {Ga_7_Co} analog by EPR. The magnetic anisotropy (zero‐field splitting, ZFS) within the *S* = 1 ground state is measured to be 4.0 cm^−1^. This is five times the magnitude of the ZFS found for {Cr_7_Mn}, which also has an *S* = 1 ground state. Hence, the large magnetic anisotropy is a direct result of the large and anisotropic Cr…Co exchange interaction in this model, and indeed neglecting these terms in the Hamiltonian gives the same value as found experimentally for {Cr_7_Mn}.

### 5.3 Proving Entanglement by 4D‐INS

**Figure 20:**
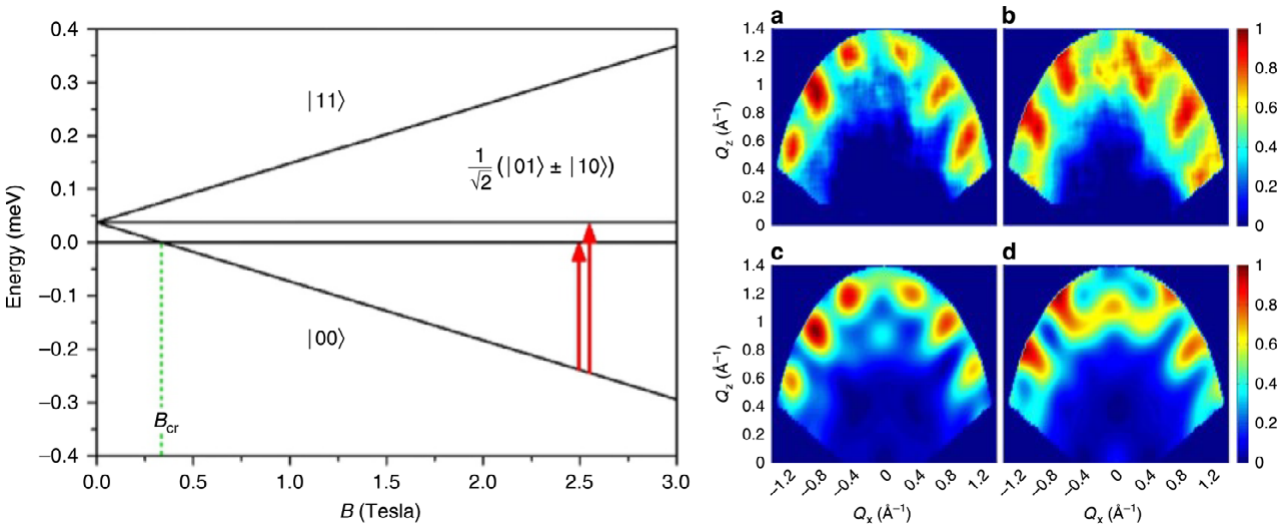
Left: singlet and triplet states of {Cr_7_Ni}_2_ dimer **50**, showing field dependence and INS transitions probed in the 4D‐INS experiment. Right: Neutron scattering intensity (7.5 Å neutrons, 1.2 K, 2.5 T applied field along *y*) as a function of momentum transfer in *xz* plane (integrated over *y*; crudely, *x* is the inter‐ring orientation, and the rings lie in the *yz* plane). (a,b) experimental data for 0.24 and 0.28 meV (1.9 and 2.3 cm^−1^) transitions, respectively; (c,d) calculated data. Figure adapted with permission from *Phys. Rev. Lett*. **2019**, *122*, 0 3702. Copyright (2019) American Physical Society.

The 4D‐INS experiment has also been used to demonstrate quantum entanglement between {Cr_7_Ni} rings in the {Cr_7_Ni}_2_ dimer [Cr_7_NiF_3_(Me‐glu) (O_2_C^t^Bu)_15_]_2_(pyrazine) **50** (Figure 20).^[^ ^71^ ^]^ Each {Cr_7_Ni} ring has a well‐isolated *S* = 1/2 ground state. Coupling of these via the short pyrazine linker gives a singlet and a triplet, which are well‐separated from all excited states; the singlet is lower energy than the triplet by ca. 0.05 meV (0.4 cm^−1^), i.e., there is an AF ring…ring coupling. At applied fields above *ca*. 0.3 T, the lowest energy component of the triplet becomes the ground state.

In the 4D‐INS experiment, the dependence of the transition intensities on the momentum transfer (**Q**), measured in 3D and as a function of energy transfer, gives direct information on the dynamical spin‐spin correlation functions.^[^ ^71^ ^]^ A magnetic field was applied to put the system into the lowest energy component (*M* = −1) of the triplet state. The excitations from this state into the *M* = 0 states of the singlet and the triplet are observed at 0.24 and 0.28 meV (1.9 and 2.3 cm^−1^). This ground state (crudely, “spin down – spin down”) is factorizable into states of the two individual rings, and therefore is not entangled. In this case, the dynamical spin‐spin correlations between pairs of spins (in different {Cr_7_Ni} rings) is nonzero only if the excited state of the transition is entangled (nonfactorizable). Such long‐distance correlations would result in modulations in the INS intensity at small *Q*. The INS was measured over a wide *Q* range, with high resolution, at 1.5 K and in 2.5 T applied field, on a large single crystal of **50** – this is a challenging experiment – and modulations at low *Q* are indeed observed for both transitions. Hence, there is entanglement in the excited states of these transitions, i.e., the *M* ═ 0 states of the singlet and triplet. The entanglement was quantified in terms of the concurrence parameter (*C*), which ranges from 0 for factorized states to 1 for maximally entangled states, by fitting to the *Q*‐dependence: this gives *C* ≈ 1 for both excited states.

### 5.4 Electric Field Effect Control of Spins

Electric (*E*) fields can be applied on much shorter length scales than magnetic fields, and for this reason electric‐field control of molecular spins is appealing. In principle, application of an external *E*‐field to a noncentrosymmetric, paramagnetic species will affect its crystal field parameters, hence energy levels and spin Hamiltonian parameters. This spin‐electric coupling can then affect EPR resonance frequencies, usually linearly for achievable *E*‐fields. Such experiments have been performed on [NH_2_Et_2_][Cr_7_MF_8_(d_9_‐O_2_C^t^Bu)_16_] with M = Ni and Mn, **1Ni** and **1Mn**, which are isostructural, noncentrosymmetric and have *S* = 1/2 and 1 ground state, respectively.^[^ ^72^ ^]^ Two electrodes are embedded in a standard EPR tube, such that a voltage (180 V) can be applied, generating an *E*‐field (in the range of 3–6 V m^−1^ for the bulk of the sample) perpendicular to the microwave *B*_1_ field. A standard Hahn echo sequence is applied, but with an *E*‐field pulse of duration *t*_E_ applied immediately after the initial π/2 microwave pulse. The echo signal is recorded as a function of *t_E_*.

**Figure 21:**
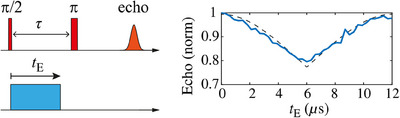
Left: *E*‐field experiment pulse sequence, involving a standard Hahn echo decay with a square *E*‐field pulse of duration *t_E_* applied immediately after the microwave pi/2 pulse. The echo is measured as a function of *t*_E_. Right: experimental echo result for d‐Cr_7_Mn (d‐toluene solution at 3 K; 180 V applied voltage; τ = 6 εs). Figure adapted from reference [65].

In the absence of the *E*‐field pulse, an echo is generated along a defined axis (perpendicular to *B*_1_) in the rotating frame. In the presence of an *E*‐field pulse, a given spin packet (group of spins with the same Larmor frequency) interacts with the *E*‐field such that its resonance frequency shifts by ∆*f*_E_ and the spins gain an additional phase during the free precession period prior to the π‐pulse. Because the measurements are performed on frozen dilute solutions, and because the sign of ∆f*_E_* is inverted with inversion of the direction of *E*, for every spin packet that is shifted by ∆*f*_E_ there is another shifted by −∆f*_E_*. The net result is a decrease in the echo amplitude. Measurements on {Cr_7_Mn} (at 3 K) indeed reveal a decrease in echo intensity with increased *E*‐field pulse duration up to *t_E_* = τ (where τ is the delay time in the Hahn echo sequence), that is, a spin‐electric coupling effect is observed (Figure 21).^[^ ^72^ ^]^ At longer *t_E_* the echo then recovers to its original intensity up to *t_E_* = 2τ: this demonstrates that the effect of the *E*‐field on the electron spins is coherent because the longer *E*‐field pulses are now refocusing the dephasing induced in the first free‐precession period. Modeling of the echo intensity as a function of the *E*‐field showed that the average induced frequency shift was about 2 ppm of the microwave frequency (9.5 GHz). Equivalent experiments on {Cr_7_Ni} showed no such modulation of the echo intensity with *t_E_*. This implies that the important factor in {Cr_7_Mn} is modulation of the ground state ZFS interaction by the *E*‐field rather than of the *g*‐values or of exchange couplings.

### 5.5 Using HMRs as a Probe of Topological Insulator Behavior

Finally, we describe an experiment where HMRs have been used as a probe to study the physical properties of another material.^[^ ^73^ ^]^ Topological insulators (TIs) are a class of material where the bulk is an electrical insulator but have conducting surface states in which the spin of the carriers is locked to their translational momentum (the origin of this effect lies in time‐reversal symmetry). The latter property means that the carriers are protected from backscattering unless the scattering centers are magnetic. The unique properties of HMRs have been exploited to perform an electrical transport experiment that probes this phenomenon by probing separately the effect on surface conductivity of a TI to closely related dia‐ and para‐magnetic molecules deposited on the surface to act as potential scatterers.

[Me_2_NH_2_][M_7_ZnF_8_(O_2_C^t^Bu)_16_] with M = Cr and Ga are paramagnetic (with an *S* = 3/2 ground state) and diamagnetic, respectively, but are isostructural, so they have near‐identical electrostatic properties. Moreover, they can be sublimed to form electrically insulating films, where electronic tunneling is not possible between the metal core of the molecules and the substrate. All of this makes them good candidates to probe electrostatic and magnetic effects on TI surface transport.

The {M_7_Zn} material was deposited on the surface of a single crystal of SmB_6_, which acts as a TI below 3 K.^[^ ^73^ ^]^ The single crystal of SmB_6_ is decorated with electrical contacts that allow both longitudinal and transverse resistance (via a voltage) to be measured with respect to an applied current. The transverse measurement is related to the Hall effect – the production of a voltage transverse to an electric current and to an applied magnetic field also perpendicular to the current. This effect is due to the Lorentz force experienced by the charge carriers due to the perpendicular field, and its magnitude is related to the carrier density (*n*). Hall resistance measurements on the pristine TI single crystal and then with a surface coating of either {Cr_7_Zn} or {Ga_7_Zn} showed identical changes (*n*_M7Zn_/*n*_pristine_ = 0.76), hence, the two surface probes induce the same change in charge carrier density. This is then solely due to the electrostatic effect of the molecules on the surface conducting state (there is no dependence on the dia/para‐magnetism). The fact that there is any change at all is evidence that it is surface conductivity that is being probed.

The longitudinal resistance measurements provide a measure of the charge carrier mobility. In nil applied field and below 3 K, {Cr_7_Zn} and {Ga_7_Zn} surface modifications are found to increase and decrease the surface state resistance, respectively. Since the Hall measurements show the carrier density to be the same, this must mean the carrier mobility is lower and higher in the presence of {Cr_7_Zn} and {Ga_7_Zn}, respectively. The ratio of the carrier mobilities (ε) in the presence of the two substrates at 1.8 K is ε_Cr7Zn_/ε_Ga7Zn_ ═ 0.84, and this must be due to the difference in magnetic properties.^[^ ^73^ ^]^

These observations confirm that these weak electrostatic and magnetic perturbations to a surface state can be independently controlled, and that paramagnetic centers are significantly more effective at scattering the charge carriers in agreement with predictions for the TI surface state. This provides an excellent example of the unexpected use of such an extensive class of molecules where the structural and magnetic properties can be varied independently and extensively.

## 6 Possible Applications of HMRs

Two distinct application areas have been studied for the HMRs.

### 6.1 Resists for Electron Beam Lithography

The constant drive of the semiconductor industry to extend Moore's Law, that is, the observation that the number of transistors per unit area doubles every two years, has led to an interest in new resist materials for lithographic processes. A colleague, Dr. Scott Lewis, had developed a program that modeled resist behavior^[^ ^74^ ^]^ and it showed that molecules with a high molecular weight but low density would show high resolution and reasonable sensitivity to reaction with electron beams. The high weight and low density combination implies molecules with a large volume, and we had a complex, which involved six {Cr_7_Ni} rings about a central {Ni_12_} ring, which met these criteria.^[^ ^46^ ^]^ High‐resolution matching large volume for the molecule may seem counter‐intuitive, but it is because the sensitivity of electron beam resists is largely driven by the number of collisions between primary or secondary electrons and the molecules. If the molecule is large, there are fewer collisions, and the reaction of the resist is largely confined to the direction defined by the original electron beam, and hence resolution is high.

Studies of [Ni_12_(chp)_12_(O_2_CMe)_6_(H_2_O)_6_{[NH_2_Pr_2_][Cr_7_NiF_8_(O_2_C^t^Bu)_15_(O_2_C‐py)] **51** confirmed that the model was correct, with 7 nm features written at 20 nm pitch.^[^ ^75^ ^]^ The etch selectivity to the plasma etching step is remarkable, with selectivity of > 30:1 against silicon in these initial studies; this allows very deep thin features to be fabricated which is relevant to the fin‐like field‐effect transistors (FIN‐FETs) now being targeted by the semiconductor industry. The etch selectivity is probably because the supramolecule decomposes to chromium oxyfluoride in the electron beam, a material known to be resistant to oxidation.

**Figure 22:**
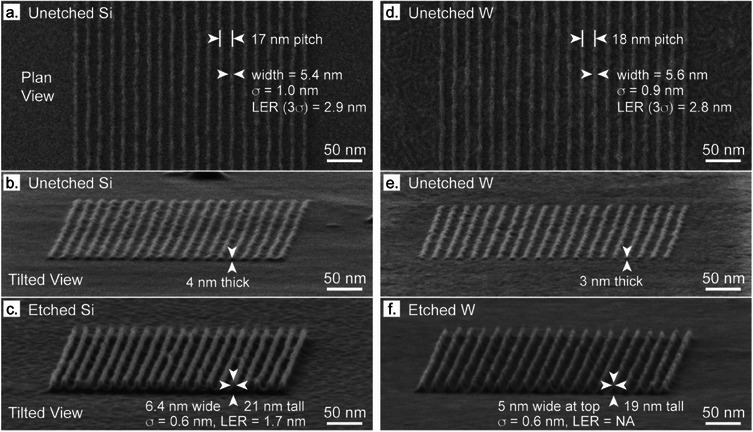
Helium ion lithography and plasma etching on **2**. (a‐c) Helium ion microscope (HIM) images of lines spaced with pitches of 17 and 18 nm on silicon substrate. (d‐f) HIM images on a 100 nm thick tungsten film. In the top row of images (a,d), developed resist structures are shown in plan view prior to a reactive ion etch. In the middle row (b,e), developed resist structures are shown when tilted to 87° prior to the etch. In the bottom row (c,f), fin‐like structures are shown following the etch. Figure reprinted with permission from *Nano Lett*. Copyright 2019 American Chemical Society^[^ ^76^ ^].^

Similar performance was found for single HRMs and for the parent homometallic [CrF(O_2_C^t^Bu)_2_]_8_ ring **2**. This can be spin coated from hexane, and using helium‐ion lithography features 5 nm wide lines on a 16 nm pitch on silicon and 6 nm lines at an 18 nm pitch on tungsten.^[^ ^76^ ^]^ This very high resolution is matched by remarkable etch selectivity: approximately 6:1 on both silicon and tungsten. This allows ca. 20 nm high structures that are ca. 5 nm wide to be fabricated from 4 nm films of **2** (Figure 22).

The ability to perform substitutions on **1** **M** structures has allowed studies of factors that affect the dose sensitivity to be investigated. [(CH_2 _= CH‐CH_2_)_2_NH_2_][Cr_7_NiF_8_(O_2_C^t^Bu)_15_(O_2_C‐Py)] **52** was synthesized, which contains diallylammonium as the cation but which is otherwise identical to **26**.^[^ ^77^ ^]^ This was then compared with [(CH_2 _= CH‐CH_2_)_2_NH_2_][Cr_7_NiF_8_(O_2_C^t^Bu)_16_] **53** and **52** bound to HgCl_2_ and HgI_2_ through nitrogen to mercury bonds. It was possible to maintain a high resolution of 16 nm half‐pitch while increasing the sensitivity of the resist to extreme‐UV radiation by a factor of 2.3 for [(**50**)HgI_2_],^[^ ^78^ ^]^ which can be attributed to the high adsorption of EUV radiation by mercury and iodine.

### 6.2 Gas Adsorption of Metal Rings

The host‐guest chemistry of the HRMs led us to examine whether we could bind small gas molecules to the rings. As gases are neutral, we began with the neutral homometallic ring **2** and studied CO_2_ uptake.^[^ ^79^ ^]^ This required desolvation of **2** by recrystallization from 1‐bromodecane. Exposure to CO_2_ at 290 K leads to adsorption of 0.8 equivalents per ring, while performing a crystal structure under a CO_2_ atmosphere leads to 100% of the central cavity. There are five contacts between 3.19 and 3.39 Å between the CO_2_ carbon and the fluorides bridging the macrocycle. DFT calculations and thermogravimetric measurements support a binding energy of around 50 kJ mol^−1^. Under the same conditions N_2_ did not bind.

Further studies showed that SO_2_ and H_2_S also bind to desolvated **2**.^[^ ^80^ ^]^ The SO_2_ binds with significant positional disorder, and O…H‐C interactions between the gas and methyl groups of pivalate ligands appear to be important in the structure. H_2_S can also be found within **2**, sitting above the plane of the metals with long S…F interactions (ca. 3.67 – 4.08 Å) which are longer than the sum of van der Waals’ radii.

## 7 Summary and Outlook

These further studies based on heterometallic {Cr^III^_x_M^II^_y_} rings and rotaxanes demonstrate that there is a great deal of new chemistry possible in this system. Specifically, the rings show a bonding to ammonium and imidazolium cations, which is distinct from the binding of organic macrocycles. Also, new rings based on {Ti^IV^_x_M^III^} cores are appearing, and they may also show different binding. Between the two families of HRMs and organic macrocycles there is scope to develop new types of host‐guest chemistry where the rings compete for binding sites, and where motion of rings between different stations in the same thread should be possible. This could lead to hybrid inorganic‐organic molecular machines.

The {Cr^III^_x_M^II^_y_} rings continue to be excellent subjects to understand the physics of antiferromagnetic coupling. Nine‐ and seven‐metal rings have now been reported, potentially allowing studies of spin frustration. The application of the {Cr^III^_7_Ni^II^} rings as qubits for quantum information processing remains a subject for study, with new proposals for algorithms and quantum error correction using these systems. For this to remain credible, a switchable gate needs to be performed. This is a massive challenge for the area, with claims of two‐qubit gates but none that involve a switch, and hence these are not yet scalable. Given there are many other potential implementation strategies, the molecular magnetism approach needs to perform switchable gates rather than proposing them. The challenges are huge, and the first such studies will likely involve switches occurring in ensembles of two‐qubit gates and not on isolated two‐qubit gates. Switching via redox chemistry has been demonstrated^[^ ^81^ ^]^ but the process is far too slow to be practical for computation.

## Conflict of Interests

The authors declare no conflict of interest.

## Data Availability

Data sharing is not applicable to this article as no new data were created or analyzed in this study.
